# Evolution of an adenocarcinoma in response to selection by targeted kinase inhibitors

**DOI:** 10.1186/gb-2010-11-8-r82

**Published:** 2010-08-09

**Authors:** Steven JM Jones, Janessa Laskin, Yvonne Y Li, Obi L Griffith, Jianghong An, Mikhail Bilenky, Yaron S Butterfield, Timothee Cezard, Eric Chuah, Richard Corbett, Anthony P Fejes, Malachi Griffith, John Yee, Montgomery Martin, Michael Mayo, Nataliya Melnyk, Ryan D Morin, Trevor J Pugh, Tesa Severson, Sohrab P Shah, Margaret Sutcliffe, Angela Tam, Jefferson Terry, Nina Thiessen, Thomas Thomson, Richard Varhol, Thomas Zeng, Yongjun Zhao, Richard A Moore, David G Huntsman, Inanc Birol, Martin Hirst, Robert A Holt, Marco A Marra

**Affiliations:** 1 Genome Sciences Centre, British Columbia Cancer Agency, 570 West 7th Avenue, Vancouver, BC, V5Z 4S6, Canada; 2 British Columbia Cancer Agency, 600 West 10th Avenue, Vancouver, BC, V5Z 4E6, Canada; 3 Vancouver General Hospital, West 12th Avenue, Vancouver, BC, V5Z 1M9, Canada; 4 Centre for Translational and Applied Genomics of British Columbia Cancer Agency and the Provincial Health Services Authority Laboratories, 600 West 10th Avenue, Vancouver, V5Z 4E6, BC, Canada; 5 Molecular Oncology, BC Cancer Research Centre, 601 West 10th Avenue, Vancouver, BC, V5Z 1L3, Canada

## Abstract

**Background:**

Adenocarcinomas of the tongue are rare and represent the minority (20 to 25%) of salivary gland tumors affecting the tongue. We investigated the utility of massively parallel sequencing to characterize an adenocarcinoma of the tongue, before and after treatment.

**Results:**

In the pre-treatment tumor we identified 7,629 genes within regions of copy number gain. There were 1,078 genes that exhibited increased expression relative to the blood and unrelated tumors and four genes contained somatic protein-coding mutations. Our analysis suggested the tumor cells were driven by the *RET *oncogene. Genes whose protein products are targeted by the RET inhibitors sunitinib and sorafenib correlated with being amplified and or highly expressed. Consistent with our observations, administration of sunitinib was associated with stable disease lasting 4 months, after which the lung lesions began to grow. Administration of sorafenib and sulindac provided disease stabilization for an additional 3 months after which the cancer progressed and new lesions appeared. A recurring metastasis possessed 7,288 genes within copy number amplicons, 385 genes exhibiting increased expression relative to other tumors and 9 new somatic protein coding mutations. The observed mutations and amplifications were consistent with therapeutic resistance arising through activation of the MAPK and AKT pathways.

**Conclusions:**

We conclude that complete genomic characterization of a rare tumor has the potential to aid in clinical decision making and identifying therapeutic approaches where no established treatment protocols exist. These results also provide direct *in vivo *genomic evidence for mutational evolution within a tumor under drug selection and potential mechanisms of drug resistance accrual.

## Background

Large-scale sequence analysis of cancer transcriptomes, predominantly using expressed sequence tags (ESTs) [1] or serial analysis of gene expression (SAGE) [2,3], has been used to identify genetic lesions that accrue during oncogenesis. Other studies have involved large-scale PCR amplification of exons and subsequent DNA sequence analysis of the amplicons to survey the mutational status of protein kinases in many cancer samples [4], 623 'cancer genes' in lung adenocarcinomas [5], 601 genes in glioblastomas, and all annotated coding sequences in breast, colorectal [6,7] and pancreatic tumors [8], searching for somatic mutations that drive oncogenesis.

The development of massively parallel sequencing technologies has provided an unprecedented opportunity to rapidly and efficiently sequence human genomes [9]. Such technology has been applied to the identification of genome rearrangements in lung cancer cell lines [10], and the sequencing of a complete acute myeloid leukemia genome [11] and a breast cancer genome [12]. The technology has also been adapted for sequencing of cancer cell line transcriptomes [13-16]. However, methodological approaches for integrated analysis of cancer genome and transcriptome sequences have not been reported; nor has there been evidence presented in the literature that such analysis has the potential to inform the choice of cancer treatment options. We present for the first time such evidence here. This approach is of particular relevance for rarer tumor types, where the scarcity of patients, their geographic distribution and the diversity of patient presentation mean that the ability to accrue sufficient patient numbers for statistically powered clinical trials is unlikely. The ability to comprehensively genetically characterize rare tumor types at an individual patient level therefore represents a logical route for informed clinical decision making and increased understanding of these diseases.

In this case the patient is a 78 year old, fit and active Caucasian man. He presented in August 2007 with throat discomfort and was found to have a 2 cm mass at the left base of the tongue. He had minimal comorbidities and no obvious risk factors for an oropharyngeal malignancy. A positron emission tomography-computed tomography (PET-CT) scan identified suspicious uptake in the primary mass and two local lymph nodes. A small biopsy of the tongue lesion revealed a papillary adenocarcinoma, although the presence in the tongue may indicate an origin in a minor salivary gland. Adenocarcinomas of the tongue are rare and represent the minority (20 to 25%) of the salivary gland tumors affecting the tongue [17-19]. In November 2007 the patient had a laser resection of the tumor and lymph node dissection. The pathology described a 1.5 cm poorly differentiated adenocarcinoma with micropapillary and mucinous features. The final surgical margins were negative. Three of 21 neck nodes (from levels 1 to 5) indicated the presence of metastatic adenocarcinoma. Subsequently, the patient received 60 Gy of adjuvant radiation therapy completed in February 2008. Four months later, although the patient remained asymptomatic, a routine follow up PET-CT scan identified numerous small (largest 1.2 cm) bilateral pulmonary metastases, none of which had been present on the pre-operative PET-CT 9 months previously. There was no evidence of local recurrence. Lacking standard chemotherapy treatment options for this rare tumor type, subsequent pathology review indicated +2 *EGFR *expression (Zymed assay) and a 6-week trial of the epidermal growth factor receptor (EGFR) inhibitor erlotinib was initiated. All the pulmonary nodules grew while on this drug, the largest lesion increasing in size from 1.5 cm to 2.1 cm from June 19th to August 18th. Chemotherapy was stopped on August 20th and a repeat CT on October 1st showed growth in all of the lung metastases. The patient provided explicit consent to pursue a genomic and transcriptome analysis and elected to undergo a fresh tumor tissue needle biopsy of a 1.7 cm left upper lobe lung lesion. This was done under CT guidance and multiple aspirates were obtained for analysis.

## Results and discussion

### DNA sequencing and mutation detection

**Figure 1:**
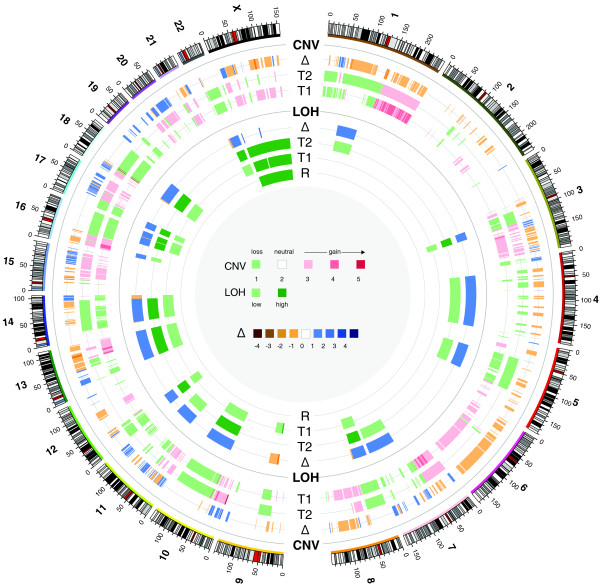
**Identified regions of chromosomal copy number variation (CNV) and loss of heterozygosity (LOH) in both the pre-treatment (T1) and post-treatment (T2) tumor samples and matched normal patient DNA (R) plotted in Circos format **[52]. CNV values are the hidden Markov model (HMM) state. Δ indicates the degree in change of HMM state between the two cancers.

There were 2,584,553,684 and 498,229,009 42-bp sequence reads that aligned to the reference human genome (HG18) from the tumor DNA and tumor transcriptome, respectively. We aligned 342,019,291 sequence reads from normal gDNA purified from peripheral blood cells and 62,517,972 sequence reads from the leukocyte transcriptome to the human reference to serve as controls. Our analysis concentrated on those genetic changes that we could predict elicited an effect on the cellular function, that is, changes in effective copy number of a gene or the sequence of a protein product. Due to our inability to usefully interpret alterations in non-coding regions, such changes were not considered. Comparison of the relative frequency of sequence alignment derived from the tumor and normal DNA identified 7,629 genes in chromosomally amplified regions, and of these, 17 genes were classified as being highly amplified. Our analysis also revealed large regions of chromosomal loss, including 12p, 17p, 18q and 22q (Figure 1). Intriguingly, we observed loss of approximately 57 megabases from 18q, although within this region we observed three highly amplified segments (Figure S3a in Additional file 1). Frequent loss of 18q has been observed in colorectal metastases. In such cases it is believed that the inactivation of the tumor suppressor protein Smad4 and the allelic loss of 18q are driving events in the formation of metastasis to the liver [20]. The expression level of Smad4 in the tumor was found to be very low (43-fold lower than in samples within our compendium of tumor expression data). Hence, down-regulation of Smad4 along with loss of 18q also appear to be properties of the tumor. Other large chromosomal losses observed in the tumor, 17p, 22q and 12p, did not correlate with losses commonly determined in previous studies of salivary gland tumors [21-23].

Our initial analysis of sequence alignments identified 84 DNA putative sequence changes corresponding to non-synonymous changes in protein coding regions present only within the tumor, of which 4 were subsequently validated to be somatic tumor mutations by Sanger sequencing (Table 1). The vast majority of false positives were due to undetected heterozygous alleles in the germline. Somatic mutations were observed in two well characterized tumor suppressor genes, *TP53 *(D259Y) and a truncating mutation in *RB1 *(L234*) removing 75% of its coding sequence [24], with *TP53 *also within a region of heterozygous loss (LOH).

**Table 1:** Predicted protein coding somatic changes within the initial and the drug resistant recurrent tumor

Tumor	Chr.	Ensembl gene ID	Ensembl display	HUGO ID	Chr. position	Ref.	Obs.	Het.	Protein position	Ref. amino acid	Alt. amino acid	Description
Initial	6	ENSG00000197062	ZNF187-201	12978	28352058	G	T	K	62	G	C	Zinc finger protein 187 (Zinc finger and SCAN domain-containing protein 26) (Protein SRE-ZBP)
Initial	8	ENSG00000169946	ZFPM2-202	16700	106884238	A	G	R	785	K	E	Zinc finger protein ZFPM2 (Zinc finger protein multitype 2) (Friend of GATA protein 2) (FOG-2) (hFOG-2)
Initial	13	ENSG00000139687	RB1-002	9884	47832247	T	A	W	234	L	*	Retinoblastoma-associated protein (pRb) (Rb) (pp110) (p105-Rb)
Initial	17	ENSG00000141510	TP53-202	11998	7518231	C	A	M	259	D	Y	Cellular tumor antigen p53 (Tumor suppressor p53) (Phosphoprotein p53) (Antigen NY-CO-13)
Recurrence	1	ENSG00000146463	ZMYM4-001	13055	35608585	G	C	S	317	Q	H	Zinc finger MYM-type protein 4 (Zinc finger protein 262)
Recurrence	2	ENSG00000118997	DNAH7-201	18661	196431742	C	G	S	2590	V	L	Dynein heavy chain 7, axonemal (Axonemal beta dynein heavy chain 7) (Ciliary dynein heavy chain 7) (Dynein heavy chain-like protein 2) (HDHC2)
Recurrence	4	ENSG00000156234	CXCL13-001	10639	78747983	G	A	R	56	R	H	C-X-C motif chemokine 13 Precursor (Small-inducible cytokine B13) (B lymphocyte chemoattractant) (CXC chemokine BLC) (B cell-attracting chemokine 1) (BCA-1) (ANGIE)
Recurrence	6	ENSG00000204228	HSD17B8-001	3554	33281235	G	A	R	141	A	T	Estradiol 17-beta-dehydrogenase 8 (EC 1.1.1.62) (Testosterone 17-beta-dehydrogenase 8) (EC 1.1.1.63) (17-beta-hydroxysteroid dehydrogenase 8) (17-beta-HSD 8) (Protein Ke6) (Ke-6)
Recurrence	7	ENSG00000186472	PCLO-201	13406	82419723	T	C	Y	2759	T	A	Protein piccolo (Aczonin)
Recurrence	11	ENSG00000152578	GRIA4-201	4574	105355581	C	T	Y	872	R	C	Glutamate receptor 4 Precursor (GluR-4) (GluR4) (GluR-D) (Glutamate receptor ionotropic, AMPA 4) (AMPA-selective glutamate receptor 4)
Recurrence	14	ENSG00000165762	OR4K2-201	14728	19414855	C	T	Y	197	L	F	Olfactory receptor 4K2 (Olfactory receptor OR14-15)
Recurrence	14	ENSG00000054654	SYNE2-206	17084	63500386	C	G	S	302	A	G	Nesprin-2 (Nuclear envelope spectrin repeat protein 2) (Synaptic nuclear envelope protein 2) (Syne-2) (Nucleus and actin connecting element protein) (Protein NUANCE)
Recurrence	18	ENSG00000173482	PTPRM-202	9675	8333477	G	A	R	929	A	T	Receptor-type tyrosine-protein phosphatase mu Precursor (Protein-tyrosine phosphatase mu) (R-PTP-mu) (EC 3.1.3.48)

Validated non-synonymous single nucleotide variations (SNVs) predicted by high-throughput sequencing are listed with the corresponding chromosome (CHr.), Ensembl gene ID, the HUGO ID, chromosomal position, the identity of the base at this location in the reference genome (Ref.), the observed base that does not match the reference (Obs.), and the IUPAC code at the heterogeneous position (Het.), the position in the protein where the amino acid changed as a result of the SNV, the reference amino acid, the altered amino acid, and the Ensembl description for this gene. Those marked as 'Initial' (first four SNVs) were identified in the primary tumor and were validated using PCR and Sanger sequencing on germline and tumor genomic DNA. Those marked as 'Recurrence' (remaining nine SNVs) were identified in the post-treatment secondary tumor and were validated by Illumina sequencing. SNVs in the initial tumor were also identified and validated in the recurrent tumor.

### Transcriptome analysis

Whole transcriptome shotgun sequencing (WTSS) [15,25] was conducted to profile the expression of tumor transcripts. In the absence of an equivalent normal tissue for comparison, we compared expression changes to the patient's leukocytes and a compendium of 50 tumor-derived WTSS datasets, which would avoid spurious observations due to technical or methodological differences between gene expression profiling platforms. This compendium approach allowed us to identify a specific and unique molecular transcript signature for this tumor, as compared to unrelated tumors, enriched in cancer causing events specific to the patient's tumor and therefore should represent relevant drug targets for therapeutic intervention. There were 3,064 differentially expressed genes (1,078 up-regulated, 1,986 down-regulated) in the lung tumor versus the blood/compendium. This analysis provided insight into those genes whose expression rate was likely to be a driving factor specific to this tumor, not identifying genes that correlate simply with proliferation and cell division. It is conceivable that such an approach, coupled with a greater understanding from multiple tumor datasets, could be replaced by the absolute quantification of oncogene expression as a means to determine clinical relevance. Changes in expression in both metastases were significantly associated with copy number changes (Figures S4 and S5 in Additional file 1). A large number of canonical pathways were identified as over-represented in the pathway analysis. Specifically, ten pathways were significant from the lung versus blood/compendium gene lists (predominantly from the down-regulated list), two from skin versus blood/compendium, and 98 from skin versus lung (predominantly over-expressed in skin relative to lung). These included many molecular mechanisms of cancer and cancer-related signaling pathways, such as mammalian target of rapamycin (mTOR) signaling, p53 signaling, Myc-mediated apoptosis signaling, vascular endothelial growth factor (VEGF) signaling, phosphoinositide 3-kinase (PI3K)/AKT signaling, and phosphatase and tensin homolog (PTEN) signaling, amongst others (Table S5 in Additional file 1).

**Figure 2:**
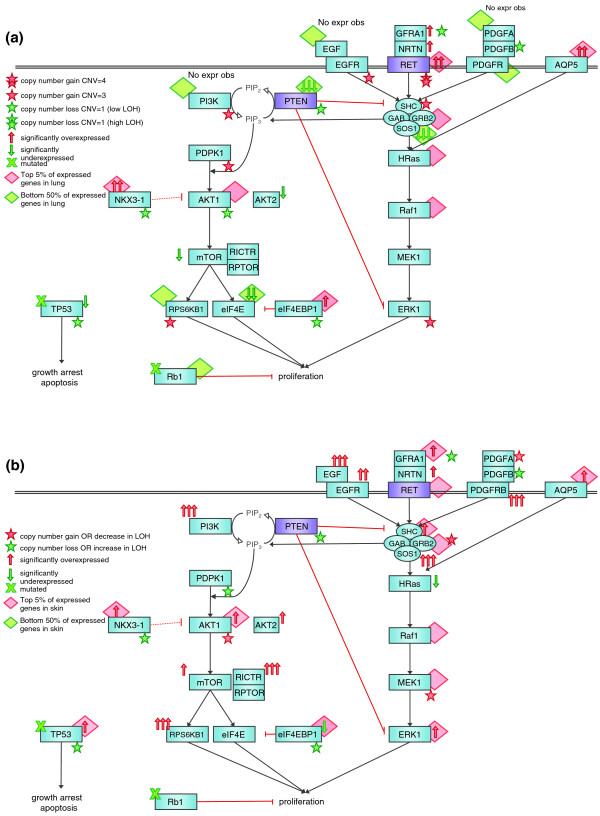
**Cancer signaling pathways affected within the tumor**. **(a) **Pre-treatment: overall, the down-regulation of *PTEN *and up-regulation of the *RET *signaling pathway appear to be driving tumor proliferation. Increased signaling independent of EGFR is consistent with the observed erlotinib insensitivity of the tumor. **(b) **Post-versus pre-treatment: after treatment with the RET inhibitors sunitinib and sorafenib, there is a marked increase in the signaling of pathway constituents, increasing tumor proliferation. Black and red pathway arrows represent activation and inhibition, respectively. Dotted arrows represent indirect interactions. The number of arrows denoting significantly over- or under-expressed genes are quantified using fold change of tumor versus compendium in (a), and primary tumor versus the tumor recurrence in (b): 1 arrow is FC ≥2; 2 arrows is FC ≥10; and 3 arrows is FC ≥50. CNV, copy number variation.

We correlated the mutated, amplified or differentially expressed genes with known cancer pathways from the Kyoto Encyclopedia of Genes and Genomes (KEGG) database [26] and to drug targets present in the DrugBank database [27]. The 15 amplified, over-expressed or mutated genes in cancer pathways targetable by approved drugs are listed in Table S2 in Additional file 1. Some amplified genes, such as *NKX3-1*, *RBBP8 *and *CABL1*, were implicated in cancer but are not well characterized in this role. In addition, they did not have known drugs targeting them. The Ret proto-oncogene (*RET*) emerged as a gene of particular interest to us, as it was present in a region of genomic amplification and was abundantly expressed. RET is a receptor tyrosine kinase that stimulates signals for cell growth and differentiation via the mitogen-activated protein kinase (MAPK)-extracellular signal-regulated kinase (ERK) pathway [28] and its constitutive activation is responsible for oncogenic transformation in medullary and papillary thyroid carcinoma [29]. In the lung tumor, *RET *was both highly amplified (hidden Markov model (HMM) level 4) and the most highly expressed known oncogene (34.5 fold change (FC) in lung relative to compendium; 123.2 FC in lung relative to blood) (Figure 2). In addition, many of the MAPK pathway constituents are also highly expressed in the tumor. Interestingly, over-expression of the water channel protein Aquaporin-5 (AQP5) has been implicated in multiple cancers and has been shown to activate Ras and its signaling pathways [30].

Aberrations leading to increased activation of the PI3K/AKT pathway are common in human cancers and are reviewed in [31]. Inactivating mutations and decreased expression (either by LOH or methylation) of *PTEN*, a tumor suppressor that reverses the action of PI3K, are the most frequently observed aberrations. In the patient tumor, *PTEN *was under-expressed (-109.7 FC in lung relative to compendium; -440.1 FC in lung relative to blood), and we note that *PTEN *maps to a region of heterozygous loss in the tumor genome. Since PTEN mediates crosstalk between PI3K and RET signaling by negatively regulating SHC and ERK [32] and up-regulated RET can also activate the PI3K/AKT pathway [33], loss of PTEN would up-regulate both the PI3K/AKT and RET-MAPK pathways, leading to decreased apoptosis, increased protein synthesis and cellular proliferation. However, in the patient, we observed LOH deletion in *AKT1*, under-expression of AKT2, mTOR, elF4E, and over-expression of the negative regulators eIF4EBP1 and NKX3-1. These changes mitigate the effect of PTEN loss on the PI3K/AKT pathway and suggest that the loss of PTEN serves primarily to further activate the RET pathway to drive tumor growth. The high expression of *RET *(which, like EGFR, activates the RAS/ERK pathway) provides a plausible explanation of the failure of erlotinib to control proliferation of this tumor. PTEN loss has also been implicated in resistance to the EGFR inhibitors gefitinib [34] and erlotinib [35], to which the tumor was determined to be insensitive. Lastly, the mutated RB1 may also play a role in the observed erlotinib insensitivity, as the loss of both RB1 and PTEN as seen in this tumor has previously been implicated in gefitinib resistance [36].

### Therapeutic intervention

**Figure 3:**
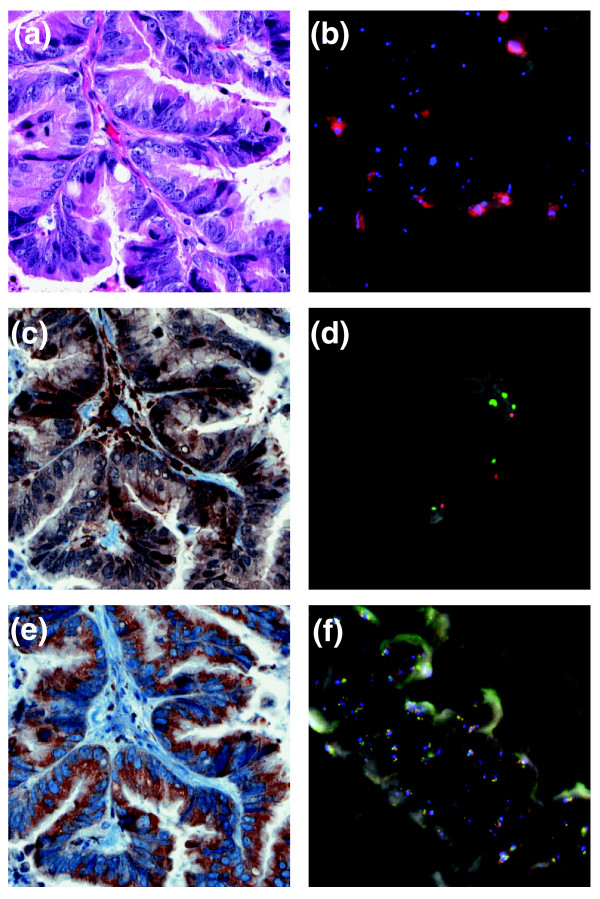
**Fluorescent *in situ *hybridization (FISH) and immunohistochemical analysis of the sublingual adenocarcinoma**. **(a) **Hematoxylin and eosin stained section of tumor (20× objective). **(b) **Striking amplification of *RBBP8 *(40×, with RBBP8 probe in red). **(c) **Focal nuclear and cytoplasmic expression of PTEN (20×) is associated with **(d) **a missing red signal indicating monoallelic loss of *PTEN *(100×; the orange gene-specific probe signals are decreased in number compared to the centromeric probe). **(e) **Diffuse, strong cytoplasmic expression of RET (20×) is associated with **(f) **amplification of the *RET *gene (40× with bacterial artificial chromosomes flanking the RET gene labeled in red and green).

**Figure 4:**
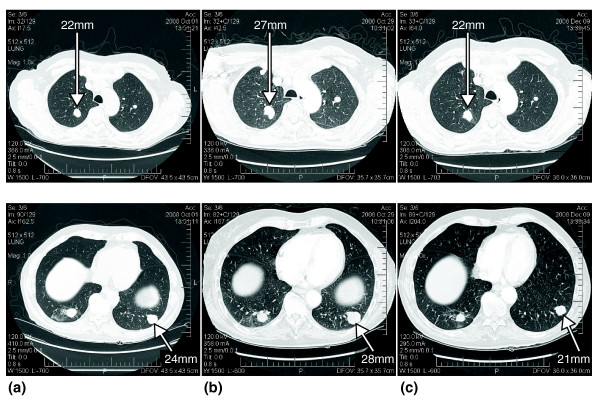
**PET-CT scans of the patient**. **(a) **1 October 2008, 1 month before sunitinib initiation. **(b) **29 October 2008, baseline before sunitinib initiation on 30 October 2008. **(c) **9 December 2008, 4 weeks on sunitinib.

The integration of copy number, expression and mutational data allowed for a compelling hypothesis of the mechanism driving the tumor and allowed identification of drugs that target the observed aberrations (Table S1 in Additional file 1). The major genomic abnormalities detected in the lung tumor sample were the up-regulation of the MAPK pathways through *RET *over-expression and *PTEN *deletion. Fluorescent *in situ *hybridization (FISH) and immunohistochemical analysis were used to confirm the status of *RET *and *PTEN *(Figure 3). Consistent with these observations, clinical administration of the RET inhibitor sunitinib had the effect of shrinking the tumors. The patient gave his full and informed consent to initiate therapy with this medication and was fully aware that adenocarcinoma of the tongue is not an approved indication for sunitinib. The drug was administered using standard dosing at 50 mg, orally, every day for 4 weeks followed by a planned 2 weeks off of the drug. After 28 days on sunitinib and 12 days off the patient had a PET-CT scan and this was compared to the baseline pretreatment scan (Figure 4). Using Response Evaluation Criteria in Solid Tumors (RECIST) criteria, the lung metastases had decreased in size by 22% and no new lesions had appeared. This was in contrast to the 16% growth seen in the previous month prior to initiation of sunitinib and the growth while on erlotinib. Because of typical side effects, his dose of sunitinib was reduced to 37.5 mg daily for 4 weeks out of 6. Repeated scanning continued to show disease stabilization and the absence of new tumor nodules for 5 months.

### Cancer recurrence

After 4 months on sunitinib, the patient's CT scan showed evidence of growth in the lung metastases. He was then switched to sorafenib and sulindac, as these were medications that were also thought to be of potential benefit given his initial genomic profiling (Table S1 in Additional file 1). Within 4 weeks a CT scan showed disease stabilization and he continued on these agents for a total of 3 months when he began to develop symptoms of disease progression. At this point he was noted to have developed recurrent disease at his primary site on the tongue, a rapidly growing skin nodule in the neck, and progressive and new lung metastases. A tumor sample was removed from the metastatic skin nodule and was subjected to both WTSS and genomic sequencing. There were 1,262,856,802 and 5,022,407,108 50-bp reads that were aligned from the transcriptome and genomic DNA, respectively. Nine new non-synonymous protein coding changes were detected that were not present within either the pre-treatment tumor or the normal DNA in addition to the four somatic changes determined in the pre-treatment tumor (Table 1). Reexamination of the sequence reads from the initial tumor analysis did not reveal the presence of any of these nine new mutated alleles even at the single read level. Extensive copy number variations were also observed in the post-treatment sample not present before treatment (Figure 1), including the arising of copy number neutral regions of LOH on chromosomes 4, 7 and 11. In the tumor recurrence, 0.13% of the genome displayed high levels of amplification, compared to 0.05% in the initial tumor sample (Table S6 in Additional file 1). Also, 24.8% of the initial tumor showed a copy number loss whereas 28.8% of the tumor recurrence showed such a loss (Table S6 in Additional file 1). We identified eight regions where the copy number status changed from a loss to a gain in the tumor recurrence and twelve regions where the copy number changed from a gain to a loss (Table S7 in Additional file 1). Indicative of heterogeneity in the tumor sample, the initial tumor showed 18.8% of the genome with incomplete LOH, whereas in the recurrence 15% of the tumor displayed an incomplete LOH signal. In the tumor recurrence 22.2% of the tumor showed a complete LOH signal, up from 5.1% in the original tumor (Table S7 Additional file 1). The previous observed pattern of focal amplification and loss of 18q in the initial tumor was recapitulated in the tumor recurrence, indicating that this specific pattern was reproducible between samples and not likely due to heterogeneity in the original tumor sample (Figure S3b in Additional file 1). There were 459 differentially expressed genes (385 up-regulated, 74 down-regulated) in the metastatic skin nodule versus the blood/compendium. Of these, 209 overlapped with the differentially expressed genes in the lung tumor versus blood/compendium set. In the skin metastasis relative to lung there were 6,440 differentially expressed genes (4,676 up-regulated, 1,764 down-regulated; Additional file 2). The 23 amplified, over-expressed or mutated genes in cancer pathways targetable by approved drugs are listed in Table S3 in Additional file 1. The cancer recurrence exhibited strong up-regulation of transcripts from genes in both the MAPK/ERK and PI3K/AKT pathways (Figure 2b). There are striking increases in expression of the receptor tyrosine kinases (EGFR, platelet-derived growth factor receptor (PDGFR)B) and their growth factor ligands (epidermal growth factor, GFRA1 (GDNF family receptor alpha 1), neurturin (NRTN)). Other genes within these pathways, such as *AKT1*, *MEK1 *and *PDGFA*, also appear amplified in copy number in the skin tumor compared to the lung tumor. Sunitinib resistance has been observed to be mediated by IL8 in renal cell carcinoma [37]. This is reflected in the tumor data, where IL8 became highly over-expressed in the cancer recurrence (FC 861.1 in skin tumor relative to lung tumor). Pathway analysis also shows IL8 signaling to be significant in the sunitinib-resistant skin tumor compared to the lung tumor (Table S6 in Additional file 1). Though the mechanism of resistance is still unclear, IL8 has been observed to transactivate EGFR and downstream ERK, stimulating cell proliferation in cancer cells [38]. Taken together, these data suggest that the mechanisms of resistance to the RET targeting selective kinase inhibitors sunitinib and sorafenib are the up-regulation of the targeted MAPK/ERK pathway and the parallel PI3K/AKT pathway. We speculate that perhaps only a cocktail of targeted drugs (that is, to RET, EGFR, mTOR, and so on) would be able to mitigate the proliferation of the tumor cells.

## Conclusions

High-throughput sequencing of the patient's tumor and normal DNA provided a comprehensive determination of copy number alterations, gene expression levels and protein coding mutations in the tumor. Correlation of the up-regulated and amplified gene products with known cancer-related pathways provided a putative mechanism of oncogenesis that was validated through the successful administration of targeted therapeutic compounds. In this case, known targets of sunitinib and sorafenib were up-regulated, implying that the tumor would be sensitive to this drug. Sequence analysis of the protein coding regions was also able to determine that the drug binding sites for sunitinib were intact. Clearly, many other changes have occurred within the tumor that likely contribute to the pathogenesis of the disease and our understanding of cancer biology is far from complete. It is possible, therefore, that these drugs may have elicited the observed clinical benefit for reasons unrelated to our hypothesis. However, this analysis did provide clinically useful information and provided the rationale for a therapeutic regime that, whilst not curative, did establish stable disease for several months. We propose that complete genetic characterization in this manner represents a tractable methodology for the study of rare cancer types and can aid in the determination of relevant therapeutic approaches in the absence of established interventions. Furthermore, the establishment of repositories containing the genomic and transcriptomic information of individual cancers coupled with their clinical responses to therapeutic intervention will be a key factor in furthering the utility of this approach. We envisage that as sequencing costs continue to decline, whole genome characterization will become a routine part of cancer pathology.

## Materials and methods

For detailed methodology see Additional file 1. A summary of the sites used for genomic and transcriptomic analyses is shown in Figure S6 in Additional file 1. Genome sequence data have been deposited at the European Genome-Phenome Archive (EGA) [39], which is hosted by the European Bioinformatics Institute (EBI), under the accession number [EBI:EGAS00000000074].

### Sample preparation

Tumor DNA was extracted from formalin-fixed, paraffin-embedded lymph node sections (slides) using the Qiagen DNeasy Blood and Tissue Kit (Qiagen, Mississauga, ON, Canada). Normal DNA was prepared from leukocytes using the Gentra PureGene blood kit as per the manufacturer's instructions (Qiagen). Genome DNA library construction and sequencing were carried out using the Genome Analyzer II (Illumina, Hayward, CA, USA) as per the manufacturer's instructions. Tumor RNA was derived from fine needle aspirates of lung metastases and normal RNA was extracted from leukocytes using Trizol (Invitrogen, Burlington, ON Canada}) and the processing for transcriptome analysis was conducted as previously described [15,16,40]. The relapse sample was obtained by surgical excision of the skin metastasis under local anesthetic 5 days after cessation with sorafenib/sulindac treatment. DNA was extracted using the Gentra PureGene Tissue kit and RNA was extracted using the Invitrogen Trizol kit, and the genomic library and transcriptome library were constructed as previously described.

### Mutation detection and copy number analysis

DNA sequences were aligned to the human reference, HG18, using MAQ version 0.7.1 [41]. To identify mutations and quantify transcript levels, WTSS data were aligned to the genome and a database of exon junctions [15]. SNPs from the tumor tissue whole genome shotgun sequencing and WTSS were detected using MAQ SNP filter parameters of consensus quality = 30 and depth = 8 and minimum mapping quality = 60. All other parameters were left as the default settings. Additional filters to reduce false positive variant calls included: the base quality score (MAQ qcal) of a variant had to be ≥20; and at least one-third of the reads at a variant position were required to possess the variant base pair. SNPs present in dbSNP [42] and established individual genomes [9,43,44] were subtracted as well as those detected in the normal patient DNA. SNPs present in the germline sample (blood) were detected using MAQ parameters at lower threshold of consensus quality = 10 and depth = 1 and minimum mapping quality = 20 in order to reduce false positive somatic mutations. Initially, non-synonymous coding SNPs were identified using Ensembl versions 49 and 50; the updated analysis presented here used version 52_36n. Candidate protein coding mutations were validated by PCR using primers using either direct Sanger sequencing or sequencing in pools on an Illumina GAiix. In the latter case, amplicons were designed such that the putative variant was located within the read length performed (75 bp). For copy number analysis, sequence quality filtering was used to remove all reads of low sequence quality (Q ≤ 10). Due to the varying amounts of sequence reads from each sample, aligned reference reads were first used to define genomic bins of equal reference coverage to which depths of alignments of sequence from each of the tumor samples were compared. This resulted in a measurement of the relative number of aligned reads from the tumors and reference in bins of variable length along the genome, where bin width is inversely proportional to the number of mapped reference reads. A HMM was used to classify and segment continuous regions of copy number loss, neutrality, or gain using methodology outlined previously [45]. The sequencing depth of the normal genome provided bins that covered over 2.9 gigabases of the HG18 reference. The five states reported by the HMM were: loss (1), neutral (2), gain (3), amplification (4), and high-level amplification (5). LOH information was generated for each sample from the lists of genomic SNPs that were identified through the MAQ pipeline. This analysis allows for classification of each SNP as either heterozygous or homozygous based on the reported SNP probabilities. For each sample, genomic bins of consistent SNP coverage are used by an HMM to identify genomic regions of consistent rates of heterozygosity. The HMM partitioned each tumor genome into three states: normal heterozygosity, increased homozygosity (low), and total homozygosity (high). We infer that a region of low homozygosity represents a state where only a portion of the cellular population had lost a copy of a chromosomal region.

### Gene expression analysis

Transcript expression was assessed at the gene level based on the total number of bases aligning to Ensembl (v52) [46] gene annotations. The corrected and normalized values for tumor gene expression (both skin and lung metastases) were then used to identify genes differentially expressed with respect to the patient's germline (blood) and a compendium of 50 previously sequenced WTSS libraries. This compendium was composed of 19 cell lines and 31 primary samples representing at least 19 different tissues and 25 tumor types as well as 6 normal or benign samples (Table S4 in Additional file 1). Tumor versus compendium comparisons used outlier statistics and tumor versus blood used Fisher's exact test. We first filtered out genes with less than 20% non-zero data across the compendium. This was necessary to avoid cases where a small expression value in the tumor receives an inflated rank when all other libraries reported zero expression (a problem common to sequencing-based expression techniques when libraries have insufficient depth). Next, we defined over-expressed genes as those with outlier and Fisher *P*-values < 0.05 and FC for tumor versus compendium and tumor versus blood > 2 and > 1.5, respectively. Similar procedures were used to define under-expressed genes. In addition to lung/skin metastasis versus compendium/normal blood we also compared the skin and lung metastases directly. Pathway analysis was performed for all gene lists using the Ingenuity Pathway Analysis software [47] (Table S5 in Additional file 1). *P*-values for differential expression and pathways analyses were corrected with the Benjamini and Hochberg method [48]. Overlaps were determined with the BioVenn web tool [49].

## Abbreviations

bp: base pair; EGFR: epidermal growth factor receptor; ERK: extracellular signal-regulated kinase; FC: fold change; HMM: hidden Markov model; IL: interleukin; LOH: loss of heterozygosity; MAPK: mitogen-activated protein kinase; mTOR: mammalian target of rapamycin; PET-CT: positron emission tomography-computed tomography; PI3K: phosphoinositide 3-kinase; PTEN: phosphatase and tensin homolog; SNP: single-nucleotide polymorphism; WTSS: whole transcriptome shotgun sequencing.

## Competing interests

The authors declare that they have no competing interests.

## Authors' contributions

SJMJ, JL, and MAM participated in experimental design, analysis and drafted the manuscript. YYL, OLG, YSB, RC and IB undertook analysis and aided in manuscript preparation. JA, MB, TC, EC, AF, MG, RDM, SPS, NT and RV contributed to the computational analysis. JY, MM, NM, MS, JT, TT, and DGH contributed to the clinical assessment of the tumor material. MM, TJP, TS, AT, TZ, YZ, RAM, MH and RAH conducted the molecular biology processing and sequencing of the clinical samples. All authors read and approved the final manuscript.

## Supplementary Material

Additional file 1 **Supplementary methods, tables and figures**. Click here for file

Additional file 2 **Supplementary expression data for identification of differentially expressed genes**. Click here for file
