# Supplementary material for: Evolution of an adenocarcinoma in response to selection by targeted kinase inhibitors
Source: Genome Biol. 2010 Aug 9;11(8):R82. doi: 10.1186/gb-2010-11-8-r82 (PMC2945784; doi:10.1186/gb-2010-11-8-r82)
Supplement: Additional file 1 — Supplementary methods, tables and figures. [file gb-2010-11-8-r82-S1.doc]

**SUPPLEMENTARY MATERIAL**

**METHODS**

**Sample Preparation**

Tumor DNA was extracted from Formalin-Fixed, Paraffin-Embedded (FFPE) lympha node sections (slides) using the Qiagen DNeasy Blood and Tissue Kit (Qiagen, Canada). The sample derived from FFPE was determined to be >80% tumor. Normal DNA was prepared from leukocytes using the Gentra PureGene blood kit as per manufacturer’s instructions (Qiagen Canada). The relapse sample was obtained by surgical excision of the skin metastasis under local anesthetic five days after cessation with sorafenib/sulindac treatment. The skin nodule removed from the patient was embedded in optimum cutting temperature (OCT) compound within 5 minutes of resection. The tissue block was frozen and transferred from the operating room in liquid nitrogen vapour before being moved to a -80 Celsius freezer for storage. Pathology review indicated that the sample collected was 99% tumor, likely the result of complete effacement of a lymph node. 150 sections were taken, 75 for DNA and 75 for RNA, which consumed less than half of the collected sample. These sections were collected in six sets of 25 sections, alternating Cell Lysis solution for DNA preparation and Trizol for RNA preparation. Between each set of 25, a section was taken for hematoxylin and eosin (H&E) staining to confirm tumor content throughout the block. All slides showed a uniform 99% tumor content with increasing tissue quantity as we sectioned towards the middle of the spherical mass. All sectioning was performed at -20 Celsius in a temperature controlled cryostat. DNA was prepared using the Gentra PureGene Tissue kit, as per manufacturers instructions (Qiagen). Total RNA was extracted using the Invitorgene Trizol kit. All RNA samples were treated with Invitrogen DNase I (amplification grade) to remove residual genomic DNA. For whole genome shotgun sequencing (WGSS) of FFPE derived tumor and normal leukocyte DNA, roughly 10 ug DNA was sheared for 10 min using Sonic Dismembrator 550 (cup horn, Fisher Scientific, Canada), and analyzed on 8% PAGE gels. A 180-220 bp DNA fraction was excised and eluted from the gel slice overnight at 4°C in 300 μl of elution buffer (5:1, LoTE buffer (3 mM Tris-HCl, pH 7.5, 0.2 mM EDTA)-7.5 M ammonium acetate), and was purified using a Spin-X Filter Tube (Fisher Scientific), and by ethanol precipitation. DNA from the relapse skin metastasis was similarly processed, although a 430-470bp DNA fraction was selected. All libraries were prepared using a paired-end protocol supplied by Illumina Inc. (USA). This involved DNA end-repair: formation of 3’ A overhangs using klenow fragment (3’ to 5’ exo minus) and ligation to Illumina PE adapters (with 5’ overhangs). Adapter-ligated products were purified on Qiaquick spin columns (Qiagen) and PCR-amplified using Phusion DNA polymerase in 10 cycles using the PE primer set (Illumina). PCR products of the desired size range were purified using 8% PAGE gels and DNA quality was assessed and quantified using an Agilent DNA 1000 series II assay and Nanodrop 7500 spectrophotometer (Nanodrop, USA) and subsequently diluted to 10nM. The final concentration was confirmed using a Quant-iT dsDNA HS assay kit and Qubit fluorometer (Invitrogen). Clusters were generated on the Illumina cluster station and paired end reads generated using an Illumina Genome Analyzer (GA2) following the manufacturer’s instructions. Image analysis, basecalling and error calibration was performed using v1.0 of Illumina’s Genome Analyzer analysis pipeline.

For whole transcriptome shotgun sequencing (WTSS) analysis methodology similar to that outlined in Morin *et al.* and Shah *et al.* [16, 40] was used. In this case, the transcriptome derived reads were aligned to the genomic sequence and also a database of known exon-exon junctions. For the tumour derived from fine needle aspirates of lung metastase, determined to be >80% tumor cells, 10ng of DNaseI treated RNA was used for the first strand synthesis followed by SMART amplification to generate double stranded cDNA. For the normeal leukocytes and the relapse skin metastasis, polyA+ RNA was purified using the MACS mRNA isolation kit (Miltenyi Biotec, Germany), from 10ug DNaseI-treated total RNA as per manufacturer’s instructions. Double-stranded cDNA was synthesized from the purified polyA^+^RNA using Superscript Double-Stranded cDNA Synthesis kit (Invitrogen, USA) and random hexamer primers (Invitrogen) at a concentration of 5µM. The double stranded cDNA was fragmented by sonication and a paired end sequencing was library prepared by following the Illumina pair end library preparation protocol described above and libraries were sequenced using the Illumina Genome Analyzer (GA2).

**Gene Expression Analysis**

Transcript expression was assessed at the gene level based on the total number of aligned bases, or observed coverage (*cvg_obs*), for all transcripts/exons at each gene locus using annotation from Ensembl (v52) [46]. The tumour transcriptome library was determined to be enriched for fragments representing contaminating genomic DNA. This effect has been observed previously with libraries constructed from very low amounts of template RNA and could be misinterpreted as gene expression. Although it should be noted that we do not know the specific cause of the increase in reads mapping to intergenic regions and it may represent a signature of a general loss of transcriptional control in the tumor cell. Nevertheless we sought to control for this effect to enable gene expression comparisons.

We compensated for this by performing a genomic subtraction. We first estimated the amount of genomic contamination in our WTSS library from the WGSS library. We have observed that WTSS libraries have between 8 and 30% intergenic (non-intronic and non-exonic) reads with a mean of ~14%. In contrast, WGSS libraries have a mean of ~58.5% intergenic reads. We used these numbers to estimate the relative amounts of WGSS and WTSS reads in the patient tumour WTSS library. We first determined the proportion of intergenic reads in the patient tumour library (~51% or 84.5 million of the mapped reads). We then formulated a simple equation to describe this library as a mixture of reads from typical WTSS and WGSS libraries where *x* is the proportion of the library that looks like a WGSS library and *y* is the proportion that looks like a WTSS library: $0.51=0.58x+0.14y$

Solving for *x* (where *x* + *y* = 1) determined that the fraction of reads derived from genomic DNA was ~0.84 based on the number of reads mapping outside of annotated gene loci. To determine the expected coverage resulting from genomic contamination (using the 84% genomic contamination value) we computed a genomic background coverage value (*cvg_gb*) from the number of exon-mapped reads in the WGSS library. For each gene (*i*), the corrected coverage (*cvg_corr*) was then computed as follows where *N_WTSS_* and *N_WGSS_* are the total number of reads in the two libraries (compensating for the fact that the genomic library was much deeper):

$$cvg\_corr_{i}=cvg\_obs_{i}-\left(cvg\_gb_{i}\times0.84\times\frac{N_{WTSS}}{N_{WGSS}}\right)$$

Adjacent normal tissue for the patient’s tumours were unavailable for differential expression analysis. Instead, the corrected and normalized values for the lung and skin metastasizes were compared to the patient’s normal blood and a reference set (‘compendium’). A direct comparison between lung and skin was also performed. The compendium was comprised of 50 other previously sequenced WTSS libraries including 19 cell lines and 31 primary samples representing at least 19 different tissues and 25 tumor types as well as 6 normal or benign samples (Table S4 in Additional file 1).

Gene expression analysis was performed with the R programming language (version 2.7.0). Coverage values (cvg_obs or cvg_corr) were scaled to the minimum total non-redundant exonic length (*L*) and then the minimum total coverage (cvg_total) as follows:

$$cvg\_scaled_{i}=\frac{\frac{cvg\_obs_{i}}{L_{i}}\times\min(L)}{cvg\_total}\times\min(cvg\_total)$$

All data were then normalized by quantiles normalization using the R quantiles function (affy library). Differential expression was calculated using outlier statistics (for tumour versus compendium) and Fisher Exact tests (for tumour versus blood or tumour versus tumour). Multiple testing correction was performed by Benjamini and Hochberg or Bonferroni method.. The dataset was first filtered to remove any genes with less than 20% non-zero data. This ensures at least some expression of the gene across the compendium. This was necessary to avoid cases where a small expression value in the tumour receives an inflated rank when all/most other libraries reported zero expression (a problem common to sequencing-based expression techniques when libraries have insufficient depth). Next, genes were removed if they did not have an Ensembl gene status of ‘KNOWN’ and a biotype of ‘protein_coding’.

Lung and skin over-expressed genes (compared to normal blood and compendium) were defined using the following criteria: (1) outlier (skin/lung vs. compendium) p-value (corrected) < 0.05; (2) Fisher (skin/lung vs. blood) p-value (corrected) < 0.05; (3) skin/lung vs. compendium FC > 2; (4) skin/lung vs. blood FC > 1.5. Lung and skin under-expressed genes were defined using the following criteria: (1) outlier (skin/lung vs. compendium) p-value (un-corrected) < 0.1; (2) Fisher (skin/lung vs. blood) p-value (corrected) < 0.05; (3) skin/lung vs. compendium FC < -2; (4) skin/lung vs. blood FC < -1.5. Finally, skin versus lung differential expression was defined using the following criteria: (1) Fisher p-value (Bonferroni corrected) < 0.001; (2a) FC > 2 (for over-expressed in skin versus lung); (2b) FC < -2 (for under-expressed in skin versus lung).

**Immunohistochemistry**

Immunohistochemistry is performed using automated methods as previously described [50], with the following antibodies: monoclonal rabbit anti-human PTEN 1:25 dilution (clone 138C6, cat# 9559, Cell Signaling Technology, Beverly, MA), goat polyclonal anti-human RET diluted 1:25 dilution (clone C-20, cat# sc-1290, Santa Cruz Biotechnology, Santa Cruz, CA), monoclonal rabbit anti-human NTRK1 1:350 dilution (clone 14G6, cat# 2508, Cell Signaling Technology), and undiluted CONFIRM anti-human EGFR (clone 3C6, cat# 790-2988, Ventana, Tucson, AZ). Hematoxylin and eosin staining is performed using standard reagents and methods.

**Fluorescence *in situ* hybridization (FISH)**

Bacterial artificial chromosomes (BACs) are obtained from the Children’s Hospital Oakland Research Institute (Oakland, CA). The BACs RP11-124O11, labelled with SpectrumRed (Abbott Molecular, Abbott Park, IL), and RP11-348I3, labelled with SpectrumGreen, flank the *RET* locus and detect disruption of *RET*. BACs RP11-66D17 (red) and RP11-1038N13 (green) flank the *NTRK1* locus and detect disruption of *NTRK1*. BAC RP11-104H10 (red) is used to detect *RBBP8* copy number. The *PTEN* and *EGFR* loci are detected with commercial probes (*EGFR*: Vysis LSI EGFR SpectrumOrange/CEP 7 SpectrumGreen probe, cat# 32-191053; *PTEN*: Vysis LSI PTEN Spectrum Orange/CEP 10 SpectrumGreen dual color probe, cat# 32-231010; Abbott Molecular). Commercial centromeric probes for chromosomes 10 and 18 are used in conjunction with the *RET* and *RBBP8* BAC probes, respectively (chr. 10: CEP 10 SpectrumAqua, cat# 32-131010; chr. 18: CEP 18 (D18Z1) SpectrumAqua, cat# 32-131018). FISH is performed as previously described [51].

**Copy number detection**

The copy number variation detection software code is available from <http://www.bcgsc.ca/platform/bioinfo/software/cnaseq>.

**Supplementary Figure S1.** The number of genes involved in each of the copy number change states.


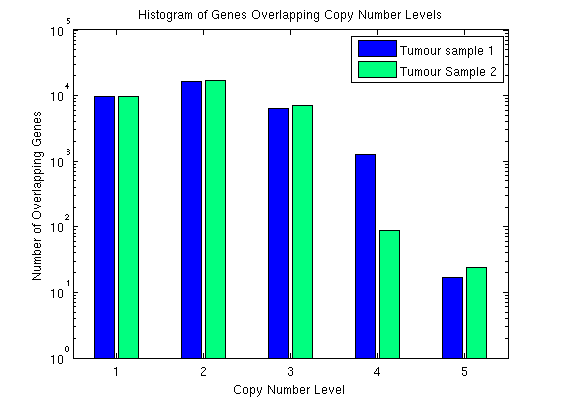


**Supplementary Figure S2.** The number of base pairs in each of the copy-number change state.


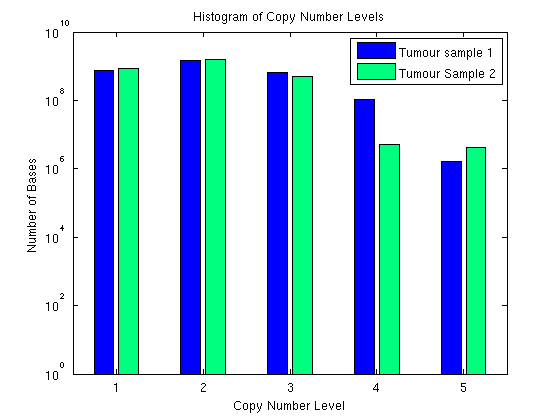


**Supplementary Figure S3**. Plots of amplification and loss states with the tumors across chromosome 18. Three discrete regions of high-amplification are observed. 16,774,297..16,943,154 (ROCK1); 18,156,373..19,144,434 (C18orf45, CTAGE1, CABLES1, RBBP8); 21,940,313..22,471,757 (TAFII105, PSMA8, KCTD1, G43631, AL831954, TAF4B). A) initial tumour B) tumor recurrence.

A.

.
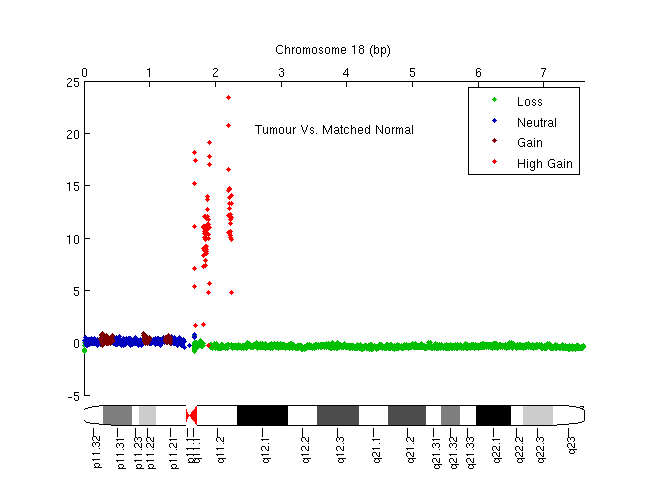


B.


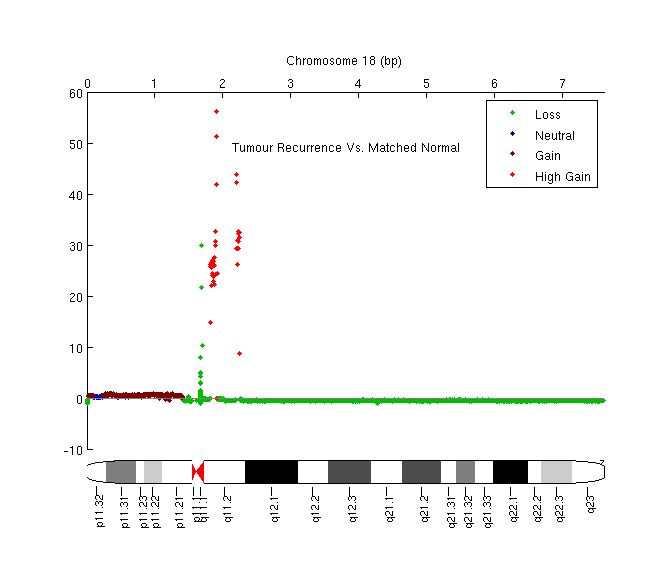


**Supplementary Figure S4**. Boxplot of copy number variation (CNV) versus differential expression value for lung metastasis relative to blood. * Indicates significant p-value for comparison to normal (CNV = 2).

**
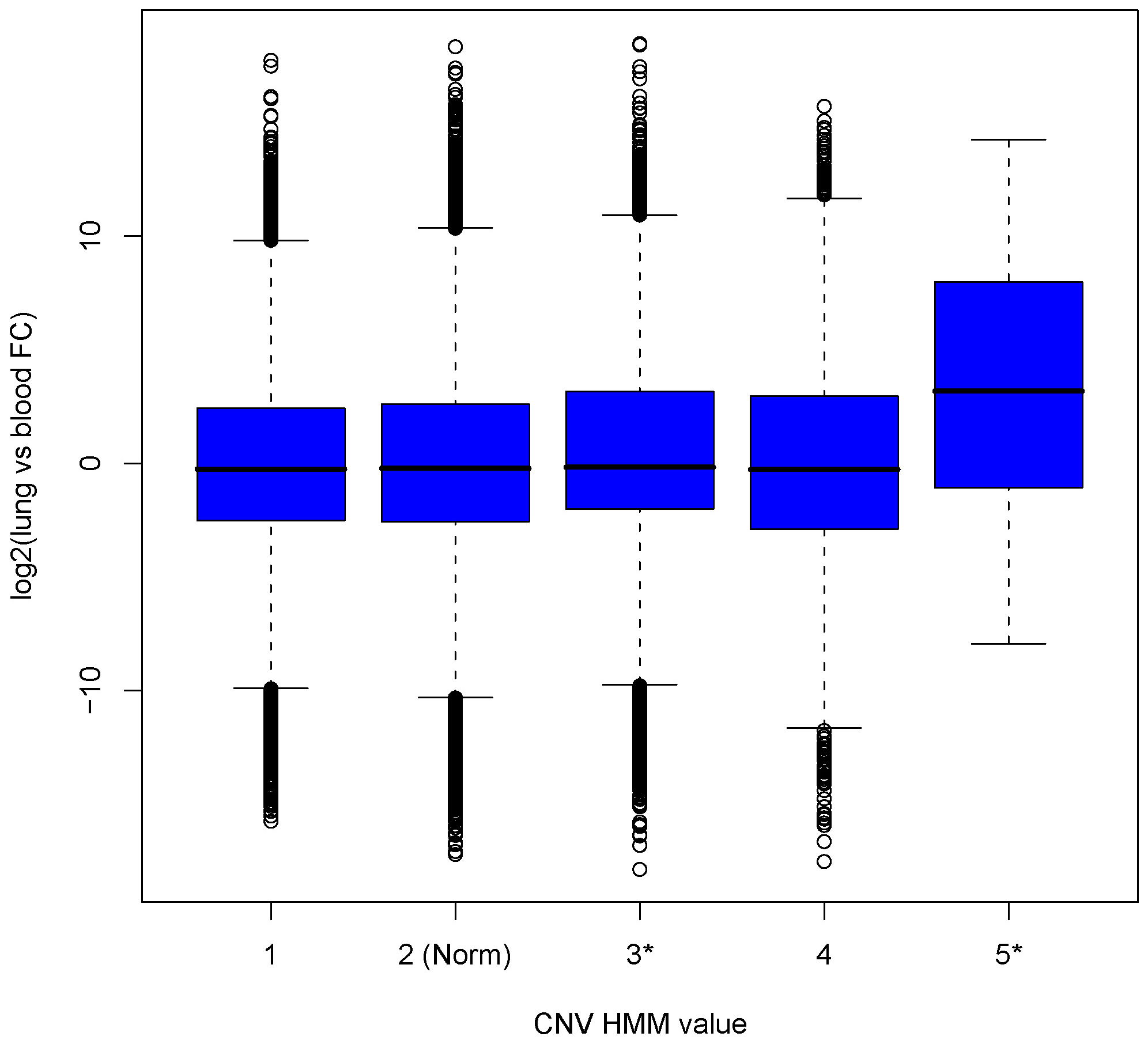
**

**Supplementary Figure S5** Boxplot of copy number variation (CNV) versus differential expression value for skin metastasis relative to blood. * Indicates significant p-value for comparison to normal (CNV = 2).

**
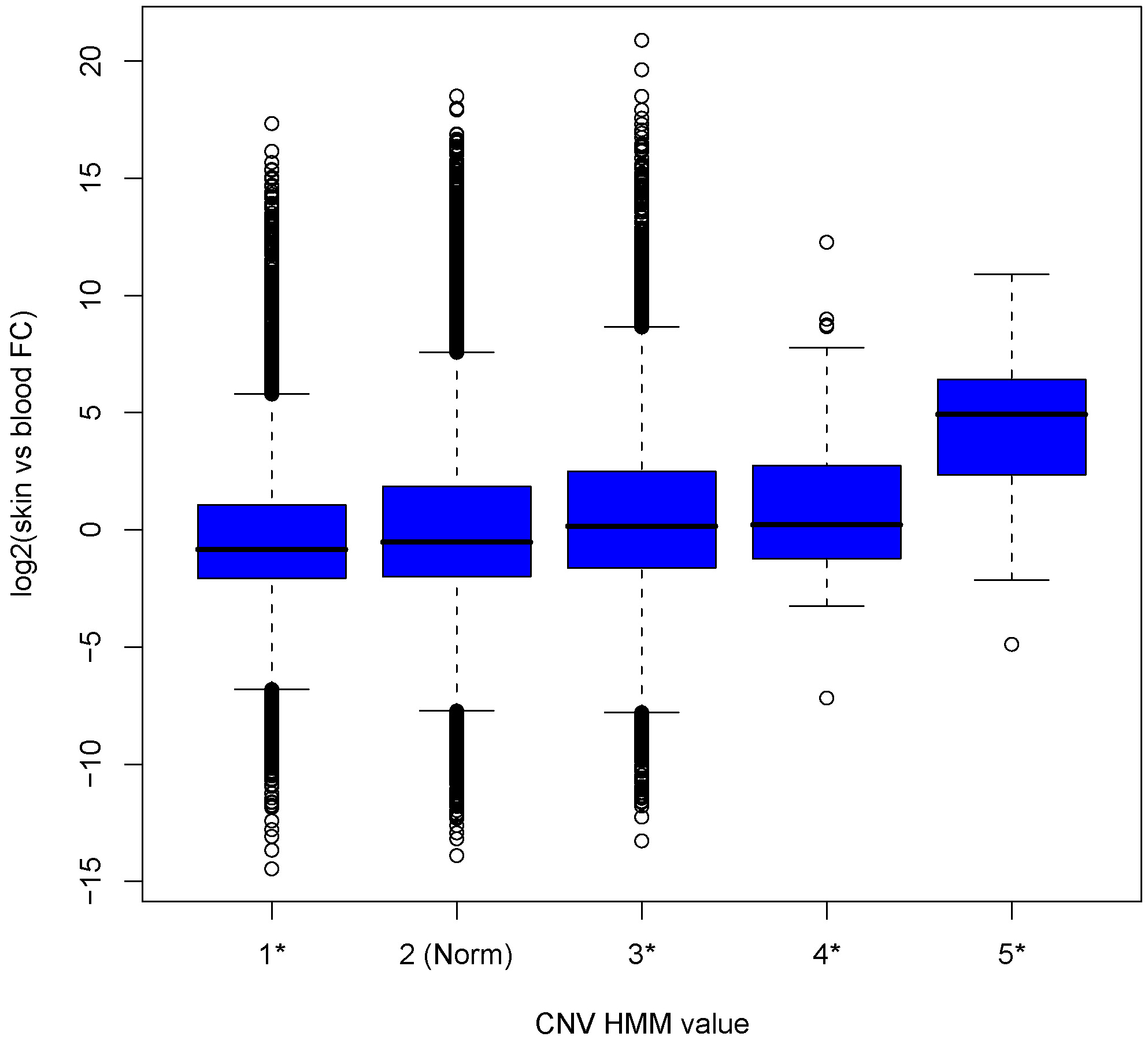
**

**Supplementary Figure S6.** Summary of sampled sites used in the genomic and transcriptomic analysis

**
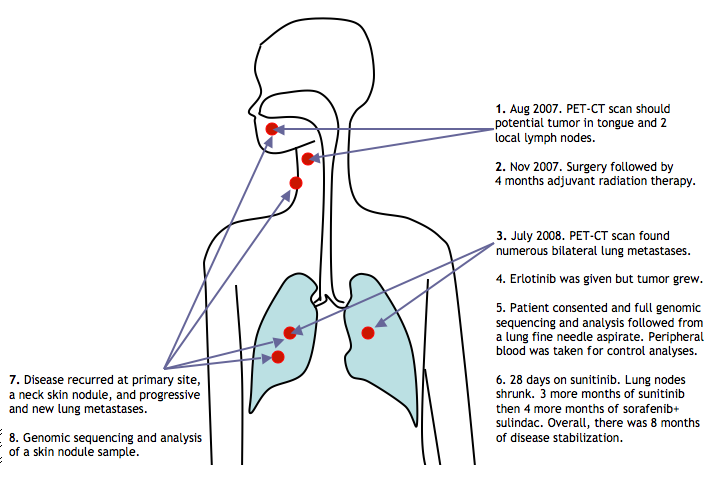
**

**Supplementary Table S1**: Potential therapeutics targeting the observed lung and skin tumor aberrations.

| Drug | Known mechanism & indications | Targeted aberrations |
| --- | --- | --- |
| Sunitinib | Targets PDGFRs, VEGFRs, RET, KIT, CSF1R, FLT3. Approved for GIST and RCC. In trials for thyroid cancer. | - Up-regulation the MAPK pathway increases cell proliferation. - RET, a validated thyroid cancer target, and its growth factors are amplified and overexpressed - AQP5 a known activator of this pathway is overexpressed - MAPK3 (ERK1) is amplified. - BRAF is also a known target in thyroid cancer. - PTEN, a suppressor of this pathway, is highly down-regulated. |
| Motesanib | Targets VEGFRs, PDGFRs, KIT, RET. In trials for thyroid cancer, GIST, NSCLC. |
| Sorafenib | Targets BRAF, RAF1, RET, VEGFRs, PDGFRB, KIT, FLT3. Approved for RCC and HCC. In trials for thyroid cancer. |
| Sulindac | An NSAID COX inhibitor for inflammation but also inhibits MAPK3 (ERK1). |

**Supplementary Table S2**: Cancer related observed lung tumour aberrations that are amplified compared to blood, significantly over expressed compared to both blood and compendium, or mutated. Only proteins that are known to be targets of approved drugs are listed. A few approved drugs known to inhibit each target are listed.

| Target | Target name | Genome aberration in lung tumour pre-treatment | Approved drug |
| --- | --- | --- | --- |
| RET | Proto-oncogene tyrosine-protein kinase receptor ret Precursor | significantly over expressed  amplified (HMM=4) | sunitinib  sorafenib |
| EGLN1 | Egl nine homolog 1 | amplified (HMM=4) | vitamin C |
| LAMC1 | Laminin subunit gamma-1 Precursor | amplified (HMM=4) | alteplase reteplase |
| PTGS2 | Prostaglandin G/H synthase 2 Precursor | amplified (HMM=4) | etoricoxib  carprofen |
| BMP2 | Bone morphogenetic protein 2 Precursor | amplified (HMM=3) | simvastatin |
| CYCS | Cytochrome c | amplified (HMM=3) | minocycline  melatonin |
| EGFR | Epidermal growth factor receptor Precursor | amplified (HMM=3) | gefitinib  erlotinib |
| GSK3B | Glycogen synthase kinase-3 beta | amplified (HMM=3) | lithium |
| HDAC2 | Histone deacetylase 2 | amplified (HMM=3) | vorinostat |
| IL6 | Interleukin-6 Precursor | amplified (HMM=3) | bicalutamide  arsenic trioxide |
| MAPK3 | Mitogen-activated protein kinase 3 | amplified (HMM=3) | sulindac  isoprotenerol |
| NTRK1 | High affinity nerve growth factor receptor Precursor | amplified (HMM=3) | imatinib |
| PRKCB | Protein kinase C beta type | amplified (HMM=3) | vitamin E |
| RAC1 | Ras-related C3 botulinum toxin substrate 1 Precursor | amplified (HMM=3) | simvastatin |
| RXRG | Retinoic acid receptor RXR-gamma | amplified (HMM=3) | tretinoin  adapalene |

**Supplementary Table S3**: Cancer related observed skin tumour aberrations that are amplified compared to blood, significantly over expressed compared to both blood and compendium, or mutated. Only proteins that are known to be targets of approved drugs are listed. A few approved drugs known to inhibit each target are listed.

| Target | Target name | Genome aberration in lung tumour pre-treatment | Approved drug |
| --- | --- | --- | --- |
| RET | Proto-oncogene tyrosine-protein kinase receptor ret Precursor | significantly over expressed  amplified (HMM=4) | sunitinib  sorafenib |
| AKT1 | RAC-alpha serine/threonine-protein kinase | significantly over expressed | arsenic trioxide |
| BMP2 | Bone morphogenetic protein 2 Precursor | amplified (HMM=3) | simvastatin |
| CYCS | Cytochrome c | amplified (HMM=3) | minocycline  melatonin |
| EGFR | Epidermal growth factor receptor Precursor | amplified (HMM=3) | gefitinib  erlotinib |
| EGLN1 | Egl nine homolog 1 | amplified (HMM=3) | vitamin C |
| ERBB2 | Receptor tyrosine-protein kinase erbB-2 Precursor | amplified (HMM=3) | lapatinib |
| GRB2 | Growth factor receptor-bound protein 2 | amplified (HMM=3) | pegademase bovine |
| GSK3B | Glycogen synthase kinase-3 beta | amplified (HMM=3) |  |
| IL6 | Interleukin-6 Precursor | amplified (HMM=3) | bicalutamide  arsenic trioxide |
| ITGA2B | Integrin alpha-IIb Precursor | amplified (HMM=3) | tirofiban |
| LAMA1 | Laminin subunit alpha-1 Precursor | amplified (HMM=3) | alteplase reteplase |
| LAMC1 | Laminin subunit gamma-1 Precursor | amplified (HMM=3) | alteplase reteplase |
| MAPK3 | Mitogen-activated protein kinase 3 | amplified (HMM=3) | sulindac  isoprotenerol |
| MMP9 | Matrix metalloproteinase-9 Precursor | amplified (HMM=3) | minocycline  simvastatin |
| NTRK1 | High affinity nerve growth factor receptor Precursor | amplified (HMM=3) | imatinib |
| PRKCA | Protein kinase C alpha type | amplified (HMM=3) | vitamin E |
| PRKCB | Protein kinase C beta type | amplified (HMM=3) | vitamin E |
| PTGS2 | Prostaglandin G/H synthase 2 Precursor | amplified (HMM=3) | etoricoxib  carprofen |
| RAC1 | Ras-related C3 botulinum toxin substrate 1 Precursor | amplified (HMM=3) | simvastatin |
| RARA | Retinoic acid receptor alpha | amplified (HMM=3) | isotretinoin  alitretinoin |
| RXRG | Retinoic acid receptor RXR-gamma | amplified (HMM=3) | tretinoin  adapalene |
| STAT5B | Signal transducer and activator of transcription 5B | amplified (HMM=3) | dasatinib |

**Supplementary Table S4 - RNA-Seq libraries included in compendium.**

Cell line names are listed in brackets under ‘Tissue’ where applicable. Otherwise, all libraries were derived from primary tumors. The compendium is comprised of 50 RNA-Seq libraries including 19 cell lines and 31 primary samples representing at least 19 different tissues and 25 tumor types as well as 6 normal or benign samples.

| **Tissue (Cell Line: If applicable)** | **Description** | **Gender** |
| --- | --- | --- |
| Bone marrow | Acute Lymphoblastic Leukemia | Unknown |
| Bone marrow | Acute Lymphoblastic Leukemia | Unknown |
| Brain | Oligodendroglioma | Unknown |
| Brain | Oligodendroglioma | Unknown |
| Brain | Oligodendroglioma | Unknown |
| Brain (NB88) | Neuroblastoma | Unknown |
| Brain/Bone Marrow (NB122L) | Neuroblastoma, stage 4, bone marrow metastases | Male |
| Brain/Bone Marrow (NB153) | Neuroblastoma, stage 4, bone marrow metastases | Unknown |
| Breast | Breast Tumor | Female |
| Breast | Breast Tumor | Female |
| Breast | Breast Tumor | Female |
| Breast (BT474-M1) | Solid, invasive ductal carcinoma | Female |
| Breast (HS-578T) | Aneuploid epithelial breast carcinoma | Female |
| Breast (SUM149) | Breast carcinoma | Female |
| Colon (HCT116) | Colon carcinoma | Male |
| Colon (MIP101) | Colon carcinoma | Male |
| Embryonic stem cells | Normal, undifferentiated | Male |
| Foreskin (FS210) | Normal | Male |
| Foreskin (FS248) | Normal | Male |
| Foreskin (FS253) | Normal skin-derived precursor cells | Male |
| Gastrointestinal Tract | Lymphoma | Female |
| Lung | Lung tumor | Female |
| Lung | Lung tumor | Female |
| Lung (PC9) | Lung adenocarcinoma | Unknown |
| Lymph nodes | Lymphoma | Male |
| Lymph nodes | Lymphoma | Male |
| Lymph nodes | Primary mediastinal B cell lymphoma | Female |
| Mononuclear blood cells | Acute Lymphoblastic Leukemia | Male |
| Mononuclear peripheral blood cells | Acute Lymphoblastic Leukemia | Male |
| Ovary | Endometroid ovarian cancer | Female |
| Ovary | High grade clear cell ovarian tumour | Female |
| Ovary | High grade serous cancer | Female |
| Ovary | Mucinous ovarian cancer | Female |
| Ovary | Small cell hypercalemic ovarian cancer | Female |
| Ovary | Granulosa cell ovarian tumour | Female |
| Ovary (BIN67) | Ovarian small cell carcinoma | Female |
| Ovary (SBOT 3.1) | Low grade serous ovarian tumour | Female |
| Pancreas (CAPAN-1) | Pancreatic adenocarcinoma | Male |
| Pelvis, Right | Epithelioid sarcoma | Unknown |
| Peripheral blood | Acute Lymphoblastic Leukemia | Unknown |
| Peripheral blood | Acute Lymphoblastic Leukemia | Unknown |
| Peritoneal effusion (SU-DHL-6) | B-cell Non-Hodgkin Lymphoma | Male |
| Pleural effusion (Karpas 1106P) | Primary Mediastinal B cell lymphoma | Female |
| Pleural effusion (KM-H2) | Hodgkin lymphoma, mixed cellulariity | Male |
| Skin (A431) | Epidermoid carcinoma | Female |
| Spleen | Lymphoma | Female |
| Thigh | Sarcoma | Unknown |
| Tonsil | Benign (CD77+ normoblasts) | Unknown |
| Tonsil | Benign (centroblast cells) | Unknown |
| Tonsil | Lymphoma | Male |

**Supplementary Table S5 - Summary of over-represented canonical pathways from Ingenuity Pathway Analysis of significant gene lists.**

| **Ingenuity Canonical Pathways** | **B-H**  **p-value** | **Molecules** |
| --- | --- | --- |
| lung vs blood/compendium: down | | |
| RAN Signaling | 0.0033 | KPNB1, KPNA3, KPNA5, KPNA4, KPNA6, TNPO1, RANBP2, KPNA1 |
| Regulation of eIF4 and p70S6K Signaling | 0.0033 | PABPC1, EIF2S3, EIF2S2, RPS6KB1, AKT2, PIK3C2A, EIF4EBP2, PPP2R5B, EIF3J, EIF4G3, EIF4A2, EIF3E, EIF2A, EIF4E, PPP2R5A, MTOR, MAPK14, EIF4G2, PIK3C3, PAIP1, SOS1, PIK3CB, PPP2R5E, EIF2C1 |
| EIF2 Signaling | 0.0087 | EIF2S3, PABPC1, EIF2S2, AKT2, PIK3C2A, EIF3J, EIF4G3, EIF4A2, EIF3E, EIF4E, EIF2A, EIF4G2, PIK3C3, SOS1, PAIP1, PIK3CB, EIF2AK2, GSK3B, EIF2C1 |
| Hypoxia Signaling in the Cardiovascular System | 0.0107 | TP53, UBE2H (includes EG:7328), UBE2M, UBE2L3, UBE2R2 (includes EG:54926), UBE2D2, ATF2, PTEN, ARNT, EP300, UBE2D4, UBE2G1, HIF1AN, ATF4, VHL, UBE2E1 |
| B Cell Receptor Signaling | 0.0214 | MAP2K4, RELA, CDC42, GSK3A, INPPL1, MAP3K4, PTEN, MAP3K10, MTOR, NFAT5, PPP3CB, MAP3K7, PPP3R1, PIK3C3, SOS1, ATF4, CHUK, GSK3B, RPS6KB1, MAP2K7, AKT2, PIK3C2A, ATF2, MAPK14, PTPN11, PIK3CB |
| mTOR Signaling | 0.0214 | NAPEPLD, PRKAB2, PPP2R5B, RPS6KA3, FKBP1A, EIF4A2, EIF4E, PRKAG1, MTOR, EIF4G2, RHOT1, PIK3C3, PRKAA1, EIF4B, RPS6KB1, AKT2, PIK3C2A, EIF4G3, EIF3J, EIF3E, PPP2R5A, RHOA, RPS6KA4, PIK3CB, PPP2R5E |
| Huntington's Disease Signaling | 0.0407 | MAP2K4, POLR2B, EP300, GNB1, MAP3K10, MTOR, POLR2A, POLR2C, SP1, PIK3C3, SOS1, ATF4, GOSR1, RASA1, TP53, AKT2, MAP2K7, HDAC8, PIK3C2A, HSPA9, IFT57, TBP, POLR2J2, BAX, NAPG, ZDHHC17, SIN3A, ATF2, RCOR1, DYNC1I2, DNAJC5, TAF4, PIK3CB, CAPN7 |
| EGF Signaling | 0.0427 | MAP2K4, CSNK2A2, MAP2K7, JAK1, PIK3C2A, PIK3C3, SOS1, SRF, PLCG1, PIK3CB, RASA1 |
| FLT3 Signaling in Hematopoietic Progenitor Cells | 0.0427 | RPS6KB1, AKT2, PIK3C2A, RPS6KA3, EIF4E, ATF2, MTOR, MAPK14, CBL, PTPN11, PIK3C3, SOS1, ATF4, PIK3CB, RPS6KA4 |
| lung vs blood/compendium: up/down | | |
| Protein Ubiquitination Pathway | 0.0000 | USP45, UBE2H (includes EG:7328), PSMA7, UBR2, UBE2L3, USP20, UBE2D2, PAN2 (includes EG:9924), BAG1, SUGT1, ANAPC11, PSMA2, USP28, UBE4B, USP38, USP27X, USP19, CBL, UBE2G1, UBE2D4, PSMB1, SMURF2, UBE2E1, VHL, ANAPC2, USP21 (includes EG:27005), USP24, USP12, USP14, PSMD9, UBR1, ANAPC10, UBE2F, UBE4A, PSMC6, STUB1, UCHL3, MED20, UBE2M, UBE2R2 (includes EG:54926), USP30, SKP1, UBE3A, USP33, XIAP, USP46, USP37 (includes EG:57695), CDC34 (includes EG:997), USP25, ANAPC1, TCEB1 |
| skin vs blood/compendium: up | | |
| O-Glycan Biosynthesis | 0.0003 | ST6GALNAC1, GALNT7, B3GNT6, GALNT5, GALNT12, GALNT6, ST3GAL4 |
| Glycosphingolipid Biosynthesis - Lactoseries | 0.0054 | FUT2, FUT6, ST3GAL4, FUT3 |
| skin vs lung: down | | |
| LXR/RXR Activation | 0.0002 | ABCG8, APOE, ECHS1, MSR1, APOC4, NR1H3, APOC2, ABCG1, IRF3, APOC1, IL1R2, LY96, NR1H2, CCL2, LCAT, LPL, PLTP, TNFRSF1B, RXRA, MMP9 |
| Natural Killer Cell Signaling | 0.0013 | RAC2, PTPN6, PRKCQ, LAIR1, TYROBP, RRAS, HRAS, INPP5D, CD300A, NCR1, LCK, MAP2K2, KLRB1, SYK, LAT, ZAP70, FCER1G, VAV1, PIK3CD, HCST, SIGLEC7, FCGR3A, PRKCB |
| LPS/IL-1 Mediated Inhibition of RXR Function | 0.0372 | ECSIT, ABCG8, APOE, APOC4, ABCG1, APOC2, CES2 (includes EG:8824), IL1R2, GSTT1, ACSL5, FABP4, CHST11, HS3ST1, FABP3, TNFRSF1B, MGMT, NR1H3, CHST12, GSTO1, APOC1, TRAF2, LY96, NR1H2, HS3ST2, NR5A2, PLTP, SLC27A3, RXRA, ABCC3, HS3ST5 |
| skin vs lung: up | | |
| Molecular Mechanisms of Cancer | 0.0000 | RAP2B, TGFBR1, PIAS2, PIK3R1, MAPK3, SUV39H1, MAP3K7IP2, SOS2, CDKN2C, GSK3A, RBL1, PAK1, GNA13, CASP10, TP53, SMAD2, AKT2, STK36, TFDP1, NFKB2, CDH1, E2F1, ARHGEF6, PRKACA, PIK3CA, CDC42, ARHGEF7, CTNNA1, PSENEN, MAP3K5 (includes EG:4217), FZD1, EP300, CHEK1, SYNGAP1, AKT1, BBC3, PIK3C3, SOS1, E2F5, PIK3R2, ARHGEF3, CASP8, CTNNB1, PRKDC, PAK2, GNAQ, MDM2, SIN3A, BAK1, APH1A (includes EG:51107), FADD, BMP6, ATR (includes EG:545), CASP7, CTNND1, MAP2K4, JAK1, PTK2, RHOB, SUFU, GSK3B, BRCA1, BIRC3, SMAD1, MAP3K7IP1, E2F2, RALGDS, CDC25A, SMAD9, CASP3, RAC1, AURKA, RAC3, PIK3R3, BMPR1B, CBL, RABIF, PTPN11, RHOA, FZD6, FZD3, PLCB3, FZD5, DIABLO, CDK2, RAP2A, RELA, PA2G4, ABL1, HIF1A, SMAD5, NFKB1, PRKAG1, CDC25B, SHC1, MAPKSP1, NLK, FANCD2, MAP3K7, ARHGEF2, CHEK2, SRC, CDC25C, PAK4, LRP5, ARHGEF12, PAK6, PIK3C2A, DVL1, PRKAR2A, GNAI1, APAF1, MAPK9, BAX, XIAP, PLCB4, PRKAR2B, MAPK14, PRKCI, FZD4, NF1, PIK3CB, ELK1, PRKAR1A |
| Rac Signaling | 0.0000 | MAP2K4, MAPK3, PIK3R1, PIKFYVE, PIP4K2B, LIMK2, PIP5K1B, PTK2, PAK1, BAIAP2, IQGAP3, RPS6KB1, RAC1, NFKB2, PLD1, PIK3R3, PIP5K1A, CYFIP1, RHOA, PIP4K2C, ELK4, RELA, ABI2, PIK3CA, CDC42, NFKB1, IQGAP1, ACTR3, ARFIP2, PIK3C3, ARPC3, PIK3R2, PI4KA, NCKAP1, ITGB1, PAK4, MAP2K7, PAK2, PAK6, PIK3C2A, ITGA2, MAP3K1, C3ORF10, ITGA3, MCF2L, PRKCI, CD44, SH3RF1, PIK3CB, ELK1 |
| p53 Signaling | 0.0001 | PIK3CA, PIK3R1, RRM2B, EP300, CHEK1, AKT1, BBC3, STAG1, PIK3C3, PPP1R13B, CCNK, SERPINB5, GSK3B, PIK3R2, CTNNB1, BRCA1, TNFRSF10A (includes EG:8797), CHEK2, TP53, PRKDC, PLAGL1, TP53INP1, AKT2, PIK3C2A, MED1, TNFRSF10B, RAC1, APAF1, CSNK1D, MDM2, BAX, RAC3, TP53BP2, PIK3R3, KAT2B, PCNA, MAPK14, E2F1, PIK3CB, SFN, ATR (includes EG:545), CDK2, SIRT1 |
| EIF2 Signaling | 0.0002 | EIF2S3, EIF2S2, PIK3CA, MAPK3, PIK3R1, SOS2, EIF1, RPS6, EIF4A2, EIF2A, SHC1, EIF2C4, AKT1, EIF4G2, PIK3C3, SOS1, EIF5, PAIP1, GSK3B, PIK3R2, EIF2C1, EIF2AK1, AKT2, PIK3C2A, EIF3H, EIF3F, RAC1, EIF3J, EIF4G3, EIF3E, RAC3, EIF2AK4, PIK3R3, EIF3C, EIF3I (includes EG:8668), PIK3CB, INSR, EIF2AK2, EIF2AK3 |
| IGF-1 Signaling | 0.0002 | IGFBP4, PIK3CA, CTGF, YWHAH, PIK3R1, MAPK3, SOS2, SRF, PRKAG1, YWHAQ (includes EG:10971), PTK2, SHC1, AKT1, PIK3C3, SOS1, CSNK2A1, PIK3R2, RPS6KB1, PXN, AKT2, YWHAG, PIK3C2A, YWHAE, RAC1, PRKAR2A, YWHAZ, RAC3, NEDD4, GRB10, PIK3R3, CSNK2A2, PRKAR2B, PRKCI, PTPN11, IGFBP3, PRKACA, PIK3CB, SFN, ELK1, CYR61, PRKAR1A |
| FAK Signaling | 0.0010 | PIK3CA, ARHGAP26, HMMR, ARHGEF7, MAPK3, PIK3R1, SOS2, ACTA2, EGF, PTK2, PAK1, AKT1, PIK3C3, SOS1, VCL, PIK3R2, EGFR, ITGB1, SRC, AKT2, PAK4, PXN, PAK2, PIK3C2A, PAK6, ACTB, ITGA2, RAC1, PLCG1, ITGA3, ACTG1, RAC3, PIK3R3, DOCK1, ARHGEF6, PIK3CB, CAPN2, CAPN7 |
| RAN Signaling | 0.0010 | KPNB1, KPNA3, KPNA6, CSE1L, KPNA2, TNPO1, RANBP2, RAN, XPO1, RCC1 (includes EG:1104), KPNA1, IPO5 |
| EGF Signaling | 0.0011 | MAP2K4, MAP2K7, PIK3CA, JAK1, PIK3C2A, PIK3R1, MAPK3, SOS2, MAP3K1, SRF, EGF, PLCG1, STAT3, PIK3R3, SHC1, CSNK2A2, PIK3C3, SOS1, CSNK2A1, PIK3CB, PIK3R2, ELK1, EGFR |
| Role of CHK Proteins in Cell Cycle Checkpoint Control | 0.0011 | TP53, CDC25C, MRE11A, RFC5, CDC2, RAD50, CHEK1, PCNA, TLK1, RFC4, E2F1, E2F5, BRCA1, ATR (includes EG:545), CHEK2, E2F2, CDK2, RFC3, CDC25A |
| Role of BRCA1 in DNA Damage Response | 0.0012 | RBBP8, FANCF, SMARCD2, MRE11A, RBL1, RAD50, CHEK1, FANCD2, E2F5, BRCA1, BLM, E2F2, CHEK2, TP53, RBL2, ATF1, FANCC, RFC5, FANCL, BACH1, MSH2, RFC4, SMARCA2, E2F1, MSH6, ATR (includes EG:545), RFC3 |
| Myc Mediated Apoptosis Signaling | 0.0012 | MAP2K4, PIK3CA, YWHAH, PIK3R1, SOS2, YWHAQ (includes EG:10971), SHC1, AKT1, PIK3C3, SOS1, PIK3R2, CASP8, TP53, AKT2, YWHAG, PIK3C2A, CASP3, YWHAE, APAF1, YWHAZ, RAC1, MAPK9, BAX, RAC3, PIK3R3, FADD, PIK3CB, SFN |
| Agrin Interactions at Neuromuscular Junction | 0.0026 | MAP2K4, CDC42, ARHGEF7, MAPK3, ACTA2, PTK2, LAMC1, PAK1, DAG1, EGFR, ITGB1, SRC, PAK4, PXN, PAK2, PAK6, ACTB, DVL1, ITGA2, RAC1, ITGA6, MAPK9, ERBB3, ITGA3, ACTG1, RAC3, ARHGEF6, GABPA, ITGA1, AGRN |
| HGF Signaling | 0.0026 | MAP2K4, PIK3CA, CDC42, PIK3R1, MAPK3, SOS2, MAP3K5 (includes EG:4217), MAP3K4, PTK2, MAP3K10, ELF4, PAK1, AKT1, MAP3K7, PIK3C3, SOS1, PIK3R2, MAP3K2, PXN, AKT2, MAP2K7, PIK3C2A, CRKL, MAP3K1, RAC1, MAPK9, PLCG1, STAT3, RAC3, ELF1, ATF2, PIK3R3, DOCK1, PRKCI, PTPN11, PIK3CB, PTGS2, ELK1, ELK3, CDK2 |
| Hypoxia Signaling in the Cardiovascular System | 0.0026 | UBE2H (includes EG:7328), UBE2L3, UBE2D2, HIF1A, EP300, ARNT, VEGFA, AKT1, HSP90AB1, ATF4, TP53, P4HB, UBE2Q1, UBE2M, UBE2R2 (includes EG:54926), NQO1, CSNK1D, BIRC6, MDM2, CREB3L4, UBE2S, ATF2, UBE2D4, UBE2G1, VHL, LDHA, UBE2E1, UBE2C, UBE2J2 |
| Cell Cycle: G2/M DNA Damage Checkpoint Regulation | 0.0031 | TP53, PRKDC, CDC25C, YWHAE, WEE1, YWHAZ, MDM2, SKP1, CDC2, SKP2, CHEK1, EP300, CDC25B, KAT2B, TOP2B, TOP2A, SFN, BRCA1, ATR (includes EG:545), CHEK2 |
| Small Cell Lung Cancer Signaling | 0.0032 | RELA, TRAF3, PIK3CA, PA2G4, PIK3R1, SUV39H1, ABL1, NFKB1, PTK2, AKT1, PIK3C3, TRAF4, PIK3R2, CHUK, TP53, AKT2, PIK3C2A, TFDP1, RAC1, APAF1, NFKB2, RAC3, SIN3A, SKP2, PIK3R3, TRAF6, E2F1, CKS1B, PIK3CB, PTGS2, CDK2 |
| ERK/MAPK Signaling | 0.0036 | YWHAH, MAPK3, PIK3R1, PPP2R5B, SOS2, SRF, PPP1R14B, PTK2, YWHAQ (includes EG:10971), PAK1, ATF4, YWHAG, CRKL, RAC1, YWHAZ, STAT3, CREB3L4, RAC3, ELF1, ATF2, PIK3R3, DUSP1, PPP2R4, PRKACA, ELK3, PIK3CA, DUSP6, RAPGEF4, PRKAG1, ELF4, SHC1, MAPKSP1, PIK3C3, SOS1, PIK3R2, ITGB1, MYCN, SRC, PXN, PAK4, PAK2, PIK3C2A, PLA2G12A, PAK6, ATF1, ITGA2, PRKAR2A, PLA2G3, PLCG1, MKNK2, ITGA3, PPP2R5A, PLA2G4A, PPP1R3D, PRKAR2B, H3F3A (includes EG:3020), PRKCI, PIK3CB, RPS6KA4, PPP2R5E, ELK1, PPP2R1B, PRKAR1A |
| IL-17 Signaling | 0.0036 | MAP2K4, RELA, PIK3CA, JAK1, MMP3, PIK3R1, MAPK3, CXCL1, IL17RC, NFKB1, TRAF3IP2, AKT1, TIMP1, MAP3K7, PIK3C3, GSK3B, PIK3R2, MAPKAPK2, IL8, AKT2, PIK3C2A, RAC1, MAPK9, RAC3, ATF2, PIK3R3, TRAF6, MAPK14, PIK3CB, PTGS2, ELK1 |
| Lymphotoxin β Receptor Signaling | 0.0036 | RELA, TRAF3, PIK3CA, PIK3R1, MAPK3, CXCL1, NFKB1, EP300, AKT1, PIK3C3, TRAF4, CHUK, PIK3R2, AKT2, CASP3, PIK3C2A, RAC1, APAF1, NFKB2, RAC3, TRAF6, PIK3R3, IKBKAP, PIK3CB, DIABLO |
| Mitotic Roles of Polo-Like Kinase | 0.0036 | ANAPC2, KIF23, CDC20, PTTG1, PPP2R5B, CDC7, ANAPC10, CDC23 (includes EG:8697), CDC25B, PLK4, HSP90AB1, CDC26, CHEK2, CDC25A, CDC25C, ESPL1, WEE1, CDC2, PPP2R5A, PPP2R4, PPP2R5E, PPP2R1B, KIF11, STAG2, CDC27, ANAPC1 |
| RAR Activation | 0.0041 | MAP2K4, TRIM24, RDH10, PIK3R1, SMARCD2, MNAT1, VEGFA, PRMT1, CSNK2A1, SMAD1, SMAD2, AKT2, SMAD9, MED1, RAC1, NFKB2, RAC3, PIK3R3, KAT2B, TAF4, SMARCA2, DUSP1, ERCC3, IGFBP3, PRKACA, NCOA1, GTF2H1, RELA, NSD1, PIK3CA, MAP3K5 (includes EG:4217), SMAD5, NFKB1, PRKAG1, EP300, AKT1, ALDH1A3, PNRC1, PIK3R2, MAPKAPK2, CITED2, MMP1 (includes EG:4312), GTF2H3, SRC, RDH11, MAP3K1, PRKAR2A, MAPK9, PARP1, CSNK2A2, PRKAR2B, MAPK14, PRKCI, CRABP2, PIK3CB, NRIP1, RBP5, CARM1, PRKAR1A |
| ERK5 Signaling | 0.0044 | IL6ST, LIF, YWHAH, RPS6KA3, EGF, YWHAQ (includes EG:10971), AKT1, ATF4, GNA13, EGFR, MAP3K2, SRC, RPS6KB1, YWHAG, LOC729991-MEF2B, YWHAE, GNAQ, YWHAZ, MEF2A (includes EG:4205), CREB3L4, ATF2, CTF1, PTPN11, MEF2D, RPS6KA4, SFN, ELK4, MAP2K5 |
| Germ Cell-Sertoli Cell Junction Signaling | 0.0044 | MAP2K4, TGFBR1, MAPK3, PIK3R1, MLLT4, LIMK2, MAP3K4, PTK2, MAP3K10, PAK1, RHOB, MTMR2, MAP3K2, PLS1, ITGA6, RAC1, RAC3, PIK3R3, CDH1, RHOA, ACTN4, CLINT1, EPN3, PIK3CA, CDC42, ACTA2, CTNNA1, MAP3K5 (includes EG:4217), IQGAP1, AKT1, WASL (includes EG:8976), MAP3K7, PIK3C3, PIK3R2, CTNNB1, ITGB1, SRC, EPN1, PXN, PAK4, MAP2K7, PAK2, PIK3C2A, PAK6, TJP1, ACTB, ITGA2, MAP3K1, MAPK9, ITGA3, ACTG1, MAPK14, PIK3CB, CTNND1 |
| AMPK Signaling | 0.0047 | CAB39, PIK3CA, PRKAB2, PRKAB1, PIK3R1, PPP2R5B, SMARCD2, PRKAG1, PPM1D, MTOR, GYS1, AKT1, PIK3C3, FASN, PRKAA1, PIK3R2, CHRNA5, SRC, RPS6KB1, AKT2, CPT1A, PIK3C2A, STRADA, AK3, RAC1, PRKAR2A, PFKP, RAC3, PFKFB2, PPP2R5A, PFKM, PIK3R3, KAT2B, MAPK14, PRKAR2B, PPM1B, PPP2R4, SMARCA2, PRKACA, AK3L1, ACACA, PIK3CB, INSR, PPP2R5E, HMGCR, AK2, PPP2R1B, PRKAR1A |
| Gα12/13 Signaling | 0.0047 | MAP2K4, RELA, PIK3CA, F2RL2, F2R, CDC42, PIK3R1, MAPK3, MAP3K5 (includes EG:4217), NFKB1, ROCK2, PTK2, AKT1, F2RL1, CDH3, PIK3C3, GNA13, CHUK, PIK3R2, CTNNB1, CDH13, VAV2, SRC, AKT2, MAP2K7, PXN, LOC729991-MEF2B, PIK3C2A, MAP3K1, RAC1, MEF2A (includes EG:4205), MAPK9, NFKB2, RAC3, LPAR3, ROCK1, PIK3R3, CDH1, LPAR1, CDH5, RHOA, MEF2D, PIK3CB, ELK1 |
| Purine Metabolism | 0.0047 | PRIM1, XDH, POLA1, RRM2B, IDE, POLM, ATP11B, BCKDHB, PRPS1, PRUNE, ATP5F1, POLR3D, GUCY1B3, RP2, AMPD3, GUCY1B2, RRM2, ENPP5, ADAR, SPAST, GART, PDE8A, PRPS2, PPP2R4, ERCC3, HPRT1, CANT1, IFNAR1, ENTPD7, ENTPD4, PDE7A, RUVBL1, WRNIP1, C22ORF30, PSMC6, PDE3B, KATNA1, ATP5J2, ENTPD3, PKM2, ATP1B1, ENPP1, PRIM2, POLR1E, POLG, PAICS, CASK, DLG3, RRM1 (includes EG:6240), ADSS, NUDT5, ADAT1, BAT1, CHRAC1, GMPS, MSH2, ATF7IP, POLA2, AK3L1, DDX1, POLH, PRTFDC1, TYMP, POLD3, DLG1, TJP2, POLR1B, POLE2, AK3, SMARCA5, PDE4B, PAPSS2, NT5C2, EIF2AK4, ENPP3, ATP5B, GUCY1A2, NP, PFAS, AK2, RFC3, POLR3F, ENTPD8, POLR2B, POLR1C, POLR2C, BLM, DHX15, ABCD3, CLPX, DHX8, HSPD1, RFC5, DDX19B, ENTPD2, NT5E, POLS, PDE8B |
| Inositol Phosphate Metabolism | 0.0093 | MAP2K4, PIK3CA, INPP1, PDIA3, MAPK3, PIK3R1, PIKFYVE, MTMR1, PIP4K2B, INPPL1, LIMK2, PIP5K1B, TTK, INPP5A, OCRL, PAK1, PRKX, CDK8, NEK2, PRPF4B, PIK3C3, PRKAA1, IP6K1, PI4K2B, PIK3R2, PLCD4, PI4KA, AKT2, IMPA1, PAK2, PIK3C2A, CSNK1D, MAPK6, MAPK9, PLCG1, CDC2, SYNJ2, PIK3R3, PLCB4, PIP5K1A, INPP5F, PLCB3, PIK3CB, EIF2AK2, ACVR2A, PIP4K2C, CDK2, DYRK1A |
| Actin Cytoskeleton Signaling | 0.0151 | F2R, MAPK3, DIAPH3, PIK3R1, SOS2, PIKFYVE, PIP4K2B, LIMK2, PIP5K1B, SSH1, PTK2, ROCK2, PAK1, BAIAP2, GNA13, SSH2, IQGAP3, CRKL, RDX, RAC1, FGD1, RAC3, PIK3R3, DOCK1, PIP5K1A, CYFIP1, RHOA, ARHGEF6, SSH3, PPP1R12B, GRLF1, ACTN4, PIP4K2C, ABI2, PIK3CA, CDC42, FGF2, ARHGEF7, ACTA2, EGF, IQGAP1, SHC1, ACTR3, WASL (includes EG:8976), PIK3C3, SOS1, ARPC3, VCL, PIK3R2, NCKAP1, ITGB1, VAV2, PXN, PAK4, PAK2, ARHGEF12, PAK6, PIK3C2A, ACTB, ITGA2, PFN2, C3ORF10, ITGA3, ACTG1, ROCK1, PIK3CB, MSN |
| Amyloid Processing | 0.0245 | AKT2, MAPK3, PRKAR2A, CSNK1D, RAC1, BACE1, PSENEN, APP, RAC3, PRKAG1, APH1A (includes EG:51107), CSNK2A2, MAPK14, AKT1, PRKAR2B, CSNK2A1, PRKACA, CAPN2, BACE2, CAPN7, GSK3B, PRKAR1A |
| CD40 Signaling | 0.0245 | MAP2K4, RELA, MAP2K7, TRAF3, PIK3CA, PIK3C2A, ATF1, MAPK3, PIK3R1, MAPK9, NFKB2, STAT3, NFKB1, TRAF6, PIK3R3, MAPK14, MAP3K7, PIK3C3, IKBKAP, PIK3CB, PIK3R2, CHUK, MAPKAPK2, MAP2K5 |
| Clathrin-mediated Endocytosis Signaling | 0.0245 | EPS15, F2R, PIK3R1, SH3GLB1, VEGFA, CD2AP, PPP3R1, RAB5C, CSNK2A1, STAM, SH3KBP1, DNM2, CLTC, USP9X, RAC1, PPP3CC, HSPA8, PIK3R3, CBL, TFRC, RAB11A, AP2A1, PIK3CA, SH3BP4, CDC42, FGF2, ACTA2, EGF, ITGB8, RAB5B, ARF6, ACTR3, WASL (includes EG:8976), PPP3CB, PIK3C3, ARPC3, LDLRAP1, PIK3R2, PPP3CA, ITGB1, AP2B1, SRC, EPN1, PIK3C2A, ACTB, MDM2, HIP1, ACTG1, CSNK2A2, LDLR, PIK3CB, MYO1E |
| Integrin Signaling | 0.0251 | RAP2B, MAP2K4, ARHGAP26, MAPK3, PIK3R1, SOS2, PTK2, TSPAN3, PAK1, RHOB, ITGAV, GSK3B, AKT2, CRKL, ITGA6, RAC1, RAC3, PIK3R3, ARHGAP5, DOCK1, ARF3, RHOA, PPP1R12B, ITGA1, CAPN2, ACTN4, CAPN7, RAP2A, PIK3CA, CDC42, ARHGEF7, ACTA2, ABL1, ITGB8, SHC1, ARF6, AKT1, ACTR3, WASL (includes EG:8976), PIK3C3, SOS1, ARPC3, VCL, PIK3R2, VASP, ITGB1, SRC, PARVA, PXN, PAK4, PAK2, PAK6, PIK3C2A, ACTB, ITGA2, PLCG1, ITGA3, ACTG1, ROCK1, ARF1, PIK3CB |
| ATM Signaling | 0.0295 | TP53, MAP2K4, CDC25C, ABL1, MAPK9, MDM2, MRE11A, CREB3L4, RAD50, CDC2, ATF2, CHEK1, FANCD2, TLK1, H2AFX, ATF4, TP53BP1, BRCA1, CHEK2, CDK2, CDC25A |
| Aldosterone Signaling in Epithelial Cells | 0.0302 | ICMT, PIK3CA, PDIA3, PIK3R1, MAPK3, SOS2, PIKFYVE, PIP4K2B, PIP5K1B, HSPA5, PIK3C3, SOS1, PIK3R2, PLCD4, PI4KA, AHCY, PIK3C2A, SLC12A2, PLCG1, NEDD4, PIK3R3, HSPA8, PLCB4, PIP5K1A, PRKCI, DUSP1, PLCB3, PIK3CB, PIP4K2C |
| Huntington's Disease Signaling | 0.0302 | MAP2K4, VTI1A, PIK3R1, SOS2, HSPA5, MAP3K10, HSPA4, ATF4, DNM2, CASP10, TP53, AKT2, CASP3, HDAC8, CLTC, HSPA9, RAC1, TBP, CREB3L4, UBE2S, NAPG, RAC3, ATF2, PIK3R3, HSPA8, DYNC1I2, HDAC3, DNAJC5, ATP5B, TAF4, PLCB3, CAPN2, GNG2, CAPN7, PIK3CA, EGF, POLR2B, EP300, SHC1, MTOR, POLR2C, AKT1, ARFIP2, SP1, PIK3C3, SOS1, PIK3R2, GOSR1, CASP8, BET1L, NAPB, EGFR, MAP2K7, PIK3C2A, YKT6, APAF1, GNAQ, MAPK9, BAX, HIP1, ZDHHC17, SIN3A, RCOR1, PLCB4, PRKCI, RCOR2, PIK3CB, CASP7 |
| Nicotinate and Nicotinamide Metabolism | 0.0302 | MAP2K4, MAPK3, NUDT12, LIMK2, TTK, VNN3, SACM1L, PRKX, PAK1, NEK2, CDK8, VNN1, PRPF4B, VNN2, ENTPD3, PRKAA1, AKT2, ENPP1, PAK2, MAPK6, CSNK1D, ENPP5, MAPK9, CDC2, NT5C2, ENPP3, NNT, NT5E, NP, EIF2AK2, NADK (includes EG:65220), ACVR2A, CDK2, DUSP16, DYRK1A |
| GNRH Signaling | 0.0355 | MAP2K4, RELA, CDC42, MAPK3, SOS2, EGF, MAP3K5 (includes EG:4217), MAP3K4, NFKB1, PRKAG1, PTK2, MAP3K10, PAK1, MAP3K7, SOS1, ATF4, DNM2, MAP3K2, EGFR, SRC, PAK4, MAP2K7, PAK2, PAK6, EGR1, MAP3K1, RAC1, GNAI1, PRKAR2A, GNAQ, MAPK9, CREB3L4, NFKB2, ATF2, PLCB4, PRKAR2B, MAPK14, PRKCI, PRKACA, PLCB3, ELK1, PRKAR1A |
| Role of PKR in Interferon Induction and Antiviral Response | 0.0389 | TP53, RELA, TRAF3, CASP3, MAP3K7IP2, APAF1, NFKB2, NFKB1, ATF2, FADD, TRAF6, AKT1, MAPK14, MAP3K7, CHUK, EIF2AK2, CASP8 |
| Nitric Oxide Signaling in the Cardiovascular System | 0.0437 | PIK3CA, PIK3R1, SLC7A1, ATP2A2, PRKAG1, VEGFA, AKT1, HSP90AB1, PDE3B, PIK3C3, PIK3R2, GUCY1B3, AKT2, CALM3, GUCY1B2, PIK3C2A, RAC1, PRKAR2A, ATP2A3, RAC3, PIK3R3, PRKAR2B, KDR, GUCY1A2, PRKACA, PIK3CB, PRKAR1A |
| Wnt/β-catenin Signaling | 0.0468 | TGFBR1, CSNK1G1, SOX12, PPP2R5B, TLE1, GSK3A, BCL9, SOX13, CSNK2A1, GSK3B, MAP3K7IP1, TP53, SOX4, AKT2, GJA1, RAC1, CSNK1D, RAC3, ACVR1B, CDH1, CDH5, PPP2R4, FZD3, FZD6, FZD5, DVL2 (includes EG:1856), FZD1, EP300, SOX9, AKT1, NLK, DKK3, CDH3, MAP3K7, CTNNB1, SRC, LRP5, PPARD, CSNK1G3, DVL1, ACVR1, GNAQ, MDM2, PPP2R5A, CSNK2A2, FZD4, CD44, DVL3, PPP2R5E, DKK1, PPP2R1B, ACVR2A |
| skin vs lung: up/down | | |
| Protein Ubiquitination Pathway | 0.0001 | UBE2H (includes EG:7328), PSMA7, UBE2L3, UBR2, UBE2D2, USP20, HSPA5, USP48, PAN2 (includes EG:9924), USP53, UBE2B, BAG1, USP10, UCHL5, SUGT1, ANAPC11, PSMA2, BRCA1, BIRC3, USP28, PSMA6, UBE2Q1, UBE4B, USP38, USP9X, PSME2, USP27X, BIRC6, USP19, PSMD3, UBE2S, NEDD4, SKP2, HSPA8, TRAF6, USP31, CBL, USP32, PSMB2, UBE2D4, UBE2G1, RBX1 (includes EG:9978), PSMB1, SMURF2, UBE2E1, VHL, UBE2J2, UBE2C, PSMB3, ANAPC2, USP21 (includes EG:27005), USP24, USP12, USP14, USP18, CDC20, PSMB10, UBR1, USP54, ANAPC10, CDC23 (includes EG:8697), UBE2F, UBE4A, USO1, UCHL1, HLA-A, PSMC6, USP47, HLA-B, MED20, UBE2M, PSMD13, UBE2R2 (includes EG:54926), MDM2, USP30, PSMA1, UBE3A, USP33, UBE2L6, SKP1, XIAP, PSME1, CUL2, UBE2E2, USP46, USP37 (includes EG:57695), CDC34 (includes EG:997), USP34 (includes EG:9736), USP25, TCEB1, ANAPC1 |
| VEGF Signaling | 0.0002 | EIF1AY, EIF2S3, RAC2, EIF2S2, PIK3CA, EIF2B4, BAD, PTK2B, MAPK3, PIK3R1, SOS2, ACTA2, EIF1, HRAS, HIF1A, ELAVL1, ARNT, ROCK2, VEGFA, PTK2, SHC1, AKT1, MAP2K2, PIK3C3, SOS1, FOXO3, PIK3R2, VCL, PXN, AKT2, VEGFB (includes EG:7423), YWHAE, PIK3C2A, RRAS, ACTB, RAC1, PLCG1, RAC3, ACTG1, ROCK1, PIK3R3, BCL2L1, KDR, PIK3CB, PIK3CD, ACTN4, SFN, PRKCB |
| Pancreatic Adenocarcinoma Signaling | 0.0002 | MAP2K4, RAC2, PLD2, JAK1, TGFBR1, BAD, SUV39H1, MAPK3, PIK3R1, CCND1, VEGFA, MAP2K2, E2F2, RALGDS, TP53, SMAD2, AKT2, PLD3, TFDP1, RAC1, HBEGF, NFKB2, STAT3, RAC3, PLD1, PIK3R3, BCL2L1, E2F1, TGFB3, PIK3CD, CDK2, RELA, PIK3CA, NAPEPLD, PA2G4, CDC42, ABL1, EGF, NFKB1, HMOX1, AKT1, PIK3C3, TGFB2, E2F5, PIK3R2, EGFR, VEGFB (includes EG:7423), PIK3C2A, MAPK9, MDM2, SIN3A, TGFA, PIK3CB, PTGS2, ELK1, MMP9 |
| Regulation of eIF4 and p70S6K Signaling | 0.0005 | EIF1AY, RAC2, EIF2S2, MAPK3, PIK3R1, PPP2R5B, SOS2, EIF1, HRAS, EIF4A2, PAIP2, EIF2A, MAP2K2, EIF4G2, PAIP1, EIF2C1, RPS6KB1, AKT2, RRAS, RAC1, EIF4G3, EIF3E, RAC3, PIK3R3, PPP2R4, EIF3C, PIK3CD, EIF2S3, PIK3CA, EIF2B4, EIF4EBP2, PPP2R2A, RPS6, EIF4EBP1, SHC1, MTOR, EIF2C4, AKT1, PIK3C3, MKNK1, SOS1, PIK3R2, ITGB1, PIK3C2A, EIF3H, EIF3F, ITGA2, EIF3J, EIF2C2, ITGA3, PPP2R5A, MAPK14, EIF3I (includes EG:8668), PIK3CB, PPP2R5E, PPP2R1B |
| PI3K/AKT Signaling | 0.0007 | RAC2, JAK1, BAD, YWHAH, MAPK3, PIK3R1, PPP2R5B, SOS2, HRAS, GSK3A, CCND1, YWHAQ (includes EG:10971), GYS1, MAP2K2, GSK3B, THEM4, TP53, RPS6KB1, AKT2, YWHAG, YWHAE, RRAS, RAC1, YWHAZ, NFKB2, RAC3, MAPK8IP1, PIK3R3, BCL2L1, PPP2R4, PIK3CD, RELA, PIK3CA, PPP2R2A, INPPL1, MAP3K5 (includes EG:4217), NFKB1, EIF4EBP1, SHC1, MTOR, AKT1, HSP90AB1, SOS1, FOXO3, HLA-B, PIK3R2, CHUK, CTNNB1, ITGB1, ITGA2, MDM2, ITGA3, INPP5D, PPP2R5A, PIK3CB, PPP2R5E, SFN, PPP2R1B |
| SAPK/JNK Signaling | 0.0008 | MAP2K4, RAC2, PIK3CA, DUSP8, CDC42, PIK3R1, SOS2, HRAS, MAP4K4, MAP3K5 (includes EG:4217), MAP3K4, HNRNPK, MAP3K10, TRADD, SHC1, LCK, GNG11, MAP3K7, DUSP10, PIK3C3, SOS1, GNA13, PIK3R2, MAP3K7IP1, MAP3K2, MAP4K2, TP53, MAP2K7, PIK3C2A, RRAS, CRKL, MAP3K1, MAPK8IP2, RAC1, MAPK9, MAPK8IP1, RAC3, ATF2, MAP4K3, PIK3R3, FADD, TRAF2, RIPK1, FCER1G, PIK3CB, PIK3CD, GNG2, ELK1 |
| Prostate Cancer Signaling | 0.0009 | RAC2, RELA, GSTP1, PIK3CA, BAD, PA2G4, PIK3R1, MAPK3, SUV39H1, SOS2, ABL1, HRAS, NFKB1, CCND1, MTOR, AKT1, HSP90AB1, MAP2K2, PIK3C3, SOS1, ATF4, GSK3B, CHUK, PIK3R2, CTNNB1, TP53, AKT2, TFDP1, PIK3C2A, RRAS, RAC1, MDM2, CREB3L4, NFKB2, RAC3, SIN3A, ATF2, PIK3R3, E2F1, NKX3-1, PIK3CB, PIK3CD, CDK2 |
| B Cell Receptor Signaling | 0.0010 | MAP2K4, RAC2, BAD, MAPK3, PIK3R1, SOS2, HRAS, GSK3A, MAP3K4, BCL6, PTPRC, MAP3K10, MAP2K2, PPP3R1, ATF4, GSK3B, MAP3K2, RPS6KB1, AKT2, PTPN6, PRKCQ, CALM3, RRAS, RAC1, NFKB2, PPP3CC, CREB3L4, RAC3, ATF2, PIK3R3, BCL2L1, PTPN11, SYK, PIK3CD, MAP2K3, VAV1, RELA, PIK3CA, CDC42, ABL1, INPPL1, MAP3K5 (includes EG:4217), NFKB1, FCGR2B, SHC1, MTOR, AKT1, NFAT5, PPP3CB, MAP3K7, PIK3C3, SOS1, CHUK, PIK3R2, PPP3CA, VAV2, MAP2K7, PIK3C2A, FCGR2A, EGR1, MAP3K1, MAPK9, INPP5D, MAPK14, DAPP1, PAG1, PIK3CB, ELK1, PRKCB |
| NF-κB Activation by Viruses | 0.0010 | RAC2, RELA, PIK3CA, PIK3R1, CD4, MAPK3, HRAS, NFKB1, LCK, AKT1, PIK3C3, ITGAV, CHUK, PIK3R2, ITGB1, AKT2, PRKCQ, PIK3C2A, RRAS, ITGA2, MAP3K1, TBP, RAC1, ITGA6, NFKB2, ITGA3, RAC3, ITGAL, TNFRSF14, PIK3R3, ITGB2, TRAF2, PRKCI, RIPK1, IKBKAP, ITGA1, PIK3CB, PIK3CD, EIF2AK2, PRKCB |
| p70S6K Signaling | 0.0010 | RAC2, PLCB2, JAK1, YWHAH, F2R, BAD, MAPK3, PIK3R1, PPP2R5B, SOS2, HRAS, YWHAQ (includes EG:10971), MAP2K2, RPS6KB1, IL4R, AKT2, PRKCQ, YWHAG, YWHAE, RRAS, EEF2, RAC1, YWHAZ, RAC3, PLD1, PIK3R3, PPP2R4, SYK, PLCB3, PIK3CD, AGTR1, PIK3CA, F2RL2, PPP2R2A, PDIA3, RPS6, SHC1, MTOR, AKT1, F2RL1, PIK3C3, SOS1, PIK3R2, EEF2K, PLCD4, EGFR, SRC, PIK3C2A, GNAI1, GNAQ, PLCG1, PPP2R5A, PLCB4, PRKCI, PIK3CB, PPP2R5E, SFN, PPP2R1B, PRKCB |
| PTEN Signaling | 0.0010 | FOXO4, RAC2, RELA, PIK3CA, CDC42, BAD, YWHAH, MAPK3, PIK3R1, SOS2, HRAS, INPPL1, GSK3A, NFKB1, CCND1, PTK2, SHC1, AKT1, MAP2K2, FOXO3, SOS1, CSNK2A1, GSK3B, CHUK, PIK3R2, EGFR, PDGFRB, ITGB1, RPS6KB1, AKT2, CASP3, RRAS, ITGA2, RAC1, NFKB2, ITGA3, RAC3, INPP5D, PIK3R3, BCL2L1, CSNK2A2, BMPR1B, GHR, CBL, PIK3CB, PIK3CD, INSR |
| TR/RXR Activation | 0.0010 | RAC2, PIK3CA, RAB3B, AKR1C3, NCOA6, PIK3R1, UCP1, HIF1A, DIO2, EP300, MTOR, AKT1, PDE3B, PIK3C3, FASN, TBL1XR1, PIK3R2, AKT2, AKR1C1, PIK3C2A, GPS2, MED1, RAC1, GRIP1, MDM2, BCL3, THRA, PFKP, AKR1C2, RAC3, DIO3, THRSP, PIK3R3, LDLR, HDAC3, COL6A3, SREBF1, ENO1, SREBF2, NCOA1, STRBP, ACACA, PIK3CB, PIK3CD, RXRA, THRB |
| Fcγ Receptor-mediated Phagocytosis in Macrophages and Monocytes | 0.0016 | RAC2, NAPEPLD, PLD2, CDC42, ARPC1B, PTK2B, MAPK3, PIK3R1, ACTA2, HMOX1, YES1, ARF6, PAK1, ACTR3, AKT1, HCK, ARPC3, PIK3R2, FGR, FCGR3A, VASP, VAV2, SRC, RPS6KB1, PXN, AKT2, PLD3, PRKCQ, FCGR2A, ACTB, RAC1, PLCG1, FYB, RAC3, ACTG1, PLD1, INPP5D, PIK3R3, DOCK1, PIP5K1A, PRKCI, CBL, ARPC2, SYK, RAB11A, VAV1, PRKCB |
| FLT3 Signaling in Hematopoietic Progenitor Cells | 0.0016 | RAC2, PIK3CA, BAD, PIK3R1, MAPK3, SOS2, RPS6KA3, HRAS, EIF4EBP1, SHC1, MTOR, AKT1, MAP2K2, PIK3C3, SOS1, ATF4, RPS6KA2, PIK3R2, STAT5A, RPS6KB1, AKT2, PIK3C2A, RRAS, RAC1, CREB3L4, STAT3, RAC3, INPP5D, ATF2, PIK3R3, MAPK14, CBL, PTPN11, PIK3CB, RPS6KA4, PIK3CD, RPS6KA1, ELK1 |
| NRF2-mediated Oxidative Stress Response | 0.0016 | MAP2K4, FTL, GSTP1, MAPK3, PIK3R1, HRAS, GCLC, SOD2, MAP2K2, ATF4, JUND, TXN, GSK3B, DNAJC16, CBR1, PRKCQ, DNAJC9, RRAS, DNAJC19, MAFF, TXNRD1, BACH1, GSR, PIK3R3, DNAJC5, ERP29, RBX1 (includes EG:9978), STIP1, DNAJC14, PIK3CD, MAP2K3, DNAJB6, MGST3, MAP2K5, FTH1, EPHX1, PIK3CA, USP14, PRDX1, NQO2, ACTA2, HSPB8, DNAJA4, DNAJC13, DNAJC10, MAP3K5 (includes EG:4217), SOD3, EP300, MAFG, CUL3, HMOX1, GSTT1, AKT1, GSTA4, DNAJC4, MAP3K7, PIK3C3, PIK3R2, FKBP5, NFE2L2, MAP2K7, SOD1, PIK3C2A, ACTB, MAP3K1, NQO1, MAPK9, SLC35A2, ACTG1, GSTO1, PRKCI, MAPK14, MGST2, GPX2, PIK3CB, CDC34 (includes EG:997), EIF2AK3, DNAJC7, PRKCB |
| Reelin Signaling in Neurons | 0.0018 | MAP2K4, PAFAH1B2, APOE, PIK3CA, PIK3R1, APP, MAP3K10, YES1, LCK, AKT1, PIK3C3, HCK, ARHGEF2, GSK3B, ARHGEF3, PIK3R2, FGR, ITGB1, SRC, MAP2K7, ARHGEF12, PIK3C2A, CRKL, MAPK8IP2, ITGA2, ITGA6, MAPK9, ITGA3, MAPK8IP1, ITGAL, PIK3R3, ITGB2, ARHGEF16, ARHGEF6, ITGA1, PIK3CB, PIK3CD, PAFAH1B1, ARHGEF9, PAFAH1B3 |
| Non-Small Cell Lung Cancer Signaling | 0.0018 | RAC2, PIK3CA, BAD, PA2G4, PIK3R1, MAPK3, SUV39H1, SOS2, ABL1, EGF, HRAS, CCND1, STK4, AKT1, MAP2K2, PIK3C3, FOXO3, SOS1, RASSF5, PIK3R2, EGFR, TP53, AKT2, PIK3C2A, TFDP1, RRAS, RAC1, PLCG1, RAC3, SIN3A, PIK3R3, E2F1, TGFA, PIK3CB, PIK3CD, RXRA |
| Leukocyte Extravasation Signaling | 0.0026 | MAP2K4, RAC2, MMP3, PIK3R1, MLLT4, MAP3K4, ROCK2, PTK2, MAP2K2, CYBB, ARHGAP12, TIMP2, PRKCQ, CRKL, RAC1, RDX, THY1, MMP2, NCF4, ARHGAP5, PIK3R3, ITGAM, CLDN12, PTPN11, CDH5, ICAM3, RHOA, NCF2, GRLF1, PIK3CD, VAV1, ACTN4, ARHGAP1, CD99 (includes EG:4267), PIK3CA, PTK2B, CDC42, MMP14, ACTA2, MMP15, ABL1, CTNNA1, RAPGEF4, WASL (includes EG:8976), MMP25, TIMP1, PIK3C3, SIPA1, PIK3R2, CLDN9, VCL, RASSF5, CTNNB1, VASP, MMP1 (includes EG:4312), ITGB1, VAV2, SRC, PXN, PIK3C2A, ACTB, GNAI1, ARHGAP4, MAPK9, PLCG1, MMP10, ACTG1, ITGAL, SELPLG, ROCK1, ITGB2, F11R, PRKCI, CLDN5, MAPK14, ARHGAP9, CD44, PIK3CB, MMP9, MSN, CTNND1, PRKCB |
| Pyrimidine Metabolism | 0.0034 | DPYSL2, POLR2F, PRIM1, POLA1, RRM2B, CTPS2, TYMP, DHODH, POLM, POLD3, MAD2L2, TXN, UNG, POLR3D, RPUSD2, TYMS, APOBEC3B, POLE4, RP2, POLR1B, CMPK2, POLE2, APOBEC2, AK3, RPUSD1, RRM2, TXNRD1, NT5C2, EIF2AK4, NME3, ENPP3, POLL, NP, TXNRD2, CANT1, ENTPD7, RFC3, POLR3F, ENTPD4, ENTPD8, DPYSL3, POLR2J, POLR2B, POLD4, POLR1C, POLR2C, ENTPD3, ENPP1, PRIM2, POLR1E, UPP1, POLG, RFC5, UMPS, RRM1 (includes EG:6240), TXNRD3, NT5C, NUDT5, CHRAC1, ITPA, NT5E, ENTPD2, EHD4, POLA2, POLS, AK3L1, UCKL1, TRUB1, POLH |
| Hereditary Breast Cancer Signaling | 0.0035 | RAC2, POLR2F, PIK3R1, FANCF, SMARCD2, HRAS, CCND1, PMS2, BRCA1, TP53, AKT2, HDAC8, RRAS, WEE1, RAC1, FANCC, RAC3, PIK3R3, PALB2, HDAC3, RFC4, SMARCA2, H2AFX, E2F1, C19ORF40, PIK3CD, NPM1 (includes EG:4869), RFC3, PIK3CA, GADD45G, BARD1, POLR2J, MRE11A, POLR2B, SMARCA4, RAD50, CHEK1, EP300, AKT1, POLR2C, FANCD2, PIK3C3, PIK3R2, BLM, CHEK2, CDC25C, PIK3C2A, RFC5, CDC2, FANCL, MSH2, MSH6, PIK3CB, SFN, ATR (includes EG:545) |
| Acute Myeloid Leukemia Signaling | 0.0036 | MAP2K4, RAC2, RELA, PIK3CA, BAD, PIK3R1, MAPK3, SOS2, HRAS, NFKB1, CCND1, EIF4EBP1, MTOR, AKT1, MAP2K2, PIM1, PIK3C3, SOS1, CEBPA, PIK3R2, CSF3R, STAT5A, RPS6KB1, AKT2, MAP2K7, PIK3C2A, RRAS, RAC1, STAT3, NFKB2, CSF1R, RAC3, PIK3R3, PIK3CB, MAP2K3, PIK3CD, MAP2K5, PIM2 (includes EG:11040) |
| Chronic Myeloid Leukemia Signaling | 0.0042 | RAC2, RELA, PIK3CA, TGFBR1, BAD, PA2G4, PIK3R1, MAPK3, SUV39H1, SOS2, ABL1, HRAS, NFKB1, RBL1, CCND1, AKT1, MAP2K2, PIK3C3, SOS1, TGFB2, E2F5, CHUK, PIK3R2, E2F2, TP53, STAT5A, RBL2, AKT2, TFDP1, PIK3C2A, HDAC8, RRAS, CRKL, RAC1, MDM2, NFKB2, RAC3, SIN3A, PIK3R3, BCL2L1, HDAC3, PTPN11, E2F1, TGFB3, PIK3CB, PIK3CD |
| Virus Entry via Endocytic Pathways | 0.0042 | RAC2, FLNB, PIK3CA, AP2A1, CDC42, PIK3R1, ACTA2, CLTB, ABL1, HRAS, ITGB8, HLA-A, FLNA, PIK3C3, HLA-B, PIK3R2, DNM2, ITGB1, SRC, AP2B1, PRKCQ, PIK3C2A, RRAS, ACTB, ITGA2, CLTC, ITGA6, RAC1, PLCG1, ITGA3, ACTG1, RAC3, ITGAL, AP2S1, FOLR1, PIK3R3, ITGB2, PRKCI, TFRC, ITGA1, PIK3CB, PIK3CD, PRKCB |
| IL-2 Signaling | 0.0056 | RAC2, PIK3CA, JAK1, PTK2B, PIK3R1, MAPK3, SOS2, HRAS, SHC1, LCK, AKT1, MAP2K2, PIK3C3, SOS1, CSNK2A1, PIK3R2, STAT5A, AKT2, PIK3C2A, RRAS, RAC1, RAC3, PIK3R3, CSNK2A2, PTPN11, SYK, PIK3CB, PIK3CD, ELK1 |
| PPARα/RXRα Activation | 0.0058 | MAP2K4, PLCB2, TGFBR1, GPD1, NCOA6, PRKAB2, PRKAB1, MAPK3, SOS2, HRAS, MAP4K4, IL1R2, MAP2K2, GPD2, LPL, PRKAA1, ADCY9, SMAD2, RRAS, MED1, NR2C2, NFKB2, MED12, ACVR1B, CAND1, TRAF6, ACADL, TGFB3, PRKACA, PLCB3, MAP2K3, INSR, ADIPOR2, RXRA, RELA, MED23, PDIA3, NFKB1, PRKAG1, EP300, SHC1, HSP90AB1, MAP3K7, FASN, SOS1, TGFB2, CLOCK, CHUK, GOT2, PLCD4, MAP2K7, ACOX1, PRKAR2A, ACVR1, CKAP5, GNAQ, PLCG1, IL1R1, AIP, PLCB4, PRKAR2B, MAPK14, GHR, ADCY7, MED24, ACVR2A, PRKAR1A, PRKCB |
| IL-8 Signaling | 0.0069 | MAP2K4, RAC2, PLD2, PLCB2, MAPK3, PIK3R1, CXCL1, HRAS, LIMK2, MAP4K4, IL8RA, CCND1, ROCK2, VEGFA, PTK2, MAP2K2, RHOB, CYBB, GNA13, RPS6KB1, IL8, AKT2, PRKCQ, PLD3, RRAS, RAC1, HBEGF, MMP2, RAC3, PLD1, PIK3R3, TRAF6, BCL2L1, CDH1, ITGAM, RHOA, NCF2, PIK3CD, GNG2, RELA, NAPEPLD, PIK3CA, PTK2B, EGF, NFKB1, EIF4EBP1, HMOX1, MTOR, AKT1, GNG11, RHOD, IL8RB, PIK3C3, PIK3R2, CHUK, EGFR, SRC, PAK2, VEGFB (includes EG:7423), PIK3C2A, GNAI1, MAPK9, BAX, ROCK1, ITGB2, PRKCI, KDR, PIK3CB, PTGS2, IRAK4, MMP9, ITGAX, PRKCB |
| Renal Cell Carcinoma Signaling | 0.0069 | RAC2, PIK3CA, CDC42, PIK3R1, MAPK3, SOS2, HRAS, HIF1A, ARNT, EP300, VEGFA, PAK1, AKT1, MAP2K2, PIK3C3, SOS1, PIK3R2, PAK4, AKT2, PAK2, PIK3C2A, PAK6, RRAS, RAC1, RAC3, PIK3R3, PTPN11, CUL2, RBX1 (includes EG:9978), TGFA, PIK3CB, PIK3CD, FH, VHL, TCEB1 |
| IL-15 Signaling | 0.0071 | RAC2, RELA, PIK3CA, JAK1, PIK3R1, MAPK3, HRAS, NFKB1, PTK2, SHC1, LCK, AKT1, MAP2K2, PIK3C3, PIK3R2, STAT5A, AKT2, PIK3C2A, RRAS, RAC1, PLCG1, NFKB2, STAT3, AXL, RAC3, PIK3R3, BCL2L1, TRAF2, MAPK14, SYK, PIK3CB, PIK3CD |
| CNTF Signaling | 0.0081 | IL6ST, PIK3CA, JAK1, CNTF, MAPK3, PIK3R1, RPS6KA3, HRAS, MTOR, AKT1, MAP2K2, PIK3C3, SOS1, PIK3R2, RPS6KA2, RPS6KB1, PIK3C2A, RRAS, STAT3, PIK3R3, PTPN11, RPS6KA4, PIK3CB, PIK3CD, RPS6KA1 |
| Endometrial Cancer Signaling | 0.0081 | RAC2, PIK3CA, BAD, PIK3R1, MAPK3, SOS2, CTNNA1, HRAS, CCND1, AKT1, MAP2K2, PIK3C3, SOS1, FOXO3, PIK3R2, GSK3B, CTNNB1, TP53, AKT2, PIK3C2A, RRAS, RAC1, RAC3, PIK3R3, CDH1, PIK3CB, PIK3CD, ELK1 |
| IL-3 Signaling | 0.0087 | RAC2, PIK3CA, JAK1, BAD, PIK3R1, MAPK3, HRAS, SHC1, PAK1, AKT1, MAP2K2, PPP3CB, PPP3R1, PIK3C3, SOS1, PIK3R2, PPP3CA, STAT5A, PTPN6, AKT2, PRKCQ, PIK3C2A, RRAS, CRKL, RAC1, PPP3CC, STAT3, RAC3, INPP5D, PIK3R3, PRKCI, PIK3CB, PIK3CD, ELK1, PRKCB |
| GM-CSF Signaling | 0.0091 | RAC2, PIK3CA, PIK3R1, MAPK3, SOS2, HRAS, CCND1, SHC1, AKT1, MAP2K2, PPP3CB, PIM1, PPP3R1, PIK3C3, SOS1, HCK, PIK3R2, PPP3CA, AKT2, PIK3C2A, RRAS, RAC1, STAT3, PPP3CC, RAC3, PIK3R3, BCL2L1, PTPN11, PIK3CB, PIK3CD, ELK1, PRKCB |
| Ovarian Cancer Signaling | 0.0091 | RAC2, SUV39H1, MAPK3, PIK3R1, HRAS, CCND1, VEGFA, MAP2K2, PMS2, GSK3B, BRCA1, TP53, RPS6KB1, AKT2, GJA1, TFDP1, RRAS, FGF9, RAC1, MMP2, RAC3, PIK3R3, E2F1, FZD3, FZD6, PRKACA, PIK3CD, FZD5, PIK3CA, PA2G4, ABL1, EGF, FZD1, WNT8B, PRKAG1, MTOR, AKT1, PIK3C3, PIK3R2, CTNNB1, EGFR, SRC, VEGFB (includes EG:7423), PIK3C2A, PRKAR2A, CGA, SIN3A, PRKAR2B, FZD4, MSH2, MSH6, CD44, PIK3CB, PTGS2, MMP9, PRKAR1A |
| Estrogen-Dependent Breast Cancer Signaling | 0.0102 | RELA, RAC2, PIK3CA, PIK3R1, MAPK3, HRAS, NFKB1, CCND1, AKT1, SP1, PIK3C3, ATF4, HSD17B12, PIK3R2, EGFR, STAT5A, SRC, AKT2, PIK3C2A, RRAS, RAC1, HSD17B7, CREB3L4, NFKB2, RAC3, ATF2, PIK3R3, PIK3CB, PIK3CD, ELK1, HSD17B14 |
| Erythropoietin Signaling | 0.0105 | RAC2, RELA, PIK3CA, PIK3R1, MAPK3, SOS2, HRAS, NFKB1, SHC1, AKT1, MAP2K2, PIK3C3, SOS1, PIK3R2, STAT5A, RPS6KB1, SRC, AKT2, PTPN6, PRKCQ, PIK3C2A, RRAS, RAC1, PLCG1, NFKB2, RAC3, PIK3R3, PRKCI, CBL, PIK3CB, PIK3CD, ELK1, PRKCB |
| Glucocorticoid Receptor Signaling | 0.0110 | RAC2, POLR2F, TAF11, TGFBR1, PIK3R1, MAPK3, SOS2, HRAS, SMARCD2, GTF2E2, MNAT1, TAF13, HMGB1 (includes EG:3146), BAG1, SMAD2, STAT5A, AKT2, MED1, RRAS, STAT3, TRAF6, HSPA8, ERCC3, PRKACA, GTF2H1, PIK3CD, PIK3CA, POLR2J, CCL5, SLPI, SMARCA4, EP300, AKT1, NCOA2, CCL2, HSP90AB1, PIK3C3, SOS1, CEBPA, TAF3, PIK3R2, FKBP5, MMP1 (includes EG:4312), PPP3CA, GTF2H3, TRAF2, SCGB1A1, GTF2E1, FKBP4, NRIP1, MAP2K4, TAF4B, JAK1, YWHAH, CD163, HSPA5, IL1R2, HSPA4, MAP2K2, PPP3R1, MAP3K7IP1, IL8, HSPA9, RAC1, TBP, PPP3CC, RAC3, MED14, PIK3R3, KAT2B, BCL2L1, TAF1, TAF4, DUSP1, SMARCA2, NCOA1, TGFB3, RELA, POLR2B, NFKB1, GTF2A1, SHC1, GTF2B, NFAT5, POLR2C, PCK2, PPP3CB, MAP3K7, ANXA1, TGFB2, CHUK, TAF2, MAP2K7, PIK3C2A, TAF15, TAF5L, MAP3K1, MAPK9, TAF6L, MAPK14, PIK3CB, NPPA, ELK1 |
| Neurotrophin/TRK Signaling | 0.0115 | MAP2K4, NTF3, PIK3CA, CDC42, PIK3R1, MAPK3, SOS2, HRAS, MAP3K5 (includes EG:4217), SHC1, AKT1, MAP2K2, PIK3C3, SOS1, ATF4, PIK3R2, FRS2, MAP2K7, PIK3C2A, SPRY1, RRAS, PLCG1, CREB3L4, ATF2, PIK3R3, PTPN11, SPRY2, PIK3CB, MAP2K3, PIK3CD, RPS6KA1, MAP2K5 |
| Renin-Angiotensin Signaling | 0.0115 | MAP2K4, RELA, PIK3CA, PTK2B, PIK3R1, MAPK3, SOS2, HRAS, CCL5, NFKB1, PRKAG1, PTK2, SHC1, PAK1, CCL2, MAP2K2, PIK3C3, SOS1, PIK3R2, ADCY9, PTPN6, PAK4, PAK2, PRKCQ, PAK6, PIK3C2A, RRAS, MAP3K1, RAC1, PRKAR2A, GNAQ, MAPK9, PLCG1, STAT3, NFKB2, ATF2, PIK3R3, MAPK14, PRKCI, PRKAR2B, PRKACA, PIK3CB, PIK3CD, ELK1, ADCY7, AGTR1, PRKAR1A, PRKCB |
| 14-3-3-mediated Signaling | 0.0126 | MAP2K4, RAC2, PLCB2, BAD, YWHAH, MAPK3, PIK3R1, HRAS, GSK3A, YWHAQ (includes EG:10971), MAP2K2, GSK3B, AKT1S1, AKT2, YWHAG, PRKCQ, YWHAE, RRAS, YWHAZ, RAC1, RAC3, PIK3R3, CBL, YAP1, PLCB3, PIK3CD, RPS6KA1, SNCA, PIK3CA, PDIA3, MAP3K5 (includes EG:4217), AKT1, TUBA8, PIK3C3, PIK3R2, PLCD4, SRC, PIK3C2A, MAPK9, PLCG1, VIM, BAX, PLCB4, TRAF2, PRKCI, PIK3CB, SFN, ELK1, PRKCB |
| HER-2 Signaling in Breast Cancer | 0.0148 | RAC2, PIK3CA, BAD, CDC42, PIK3R1, SOS2, EGF, HRAS, GSK3A, MAP3K5 (includes EG:4217), ITGB8, CCND1, AKT1, PIK3C3, SOS1, GSK3B, PIK3R2, EGFR, TP53, ITGB1, AKT2, PRKCQ, PIK3C2A, RRAS, RAC1, PLCG1, MMP2, ERBB3, MDM2, RAC3, PIK3R3, ITGB2, PRKCI, PIK3CB, PIK3CD, PRKCB |
| mTOR Signaling | 0.0151 | RAC2, PLD2, PRKAB2, PRKAB1, MAPK3, PIK3R1, PPP2R5B, HRAS, EIF4A2, VEGFA, RHOB, EIF4G2, PRKAA1, EIF4B, RPS6KB1, AKT2, PRKCQ, PLD3, RRAS, RAC1, EIF4G3, EIF3E, RAC3, PLD1, PIK3R3, PPP2R4, EIF3C, RHOA, PIK3CD, RPS6KA1, INSR, NAPEPLD, PIK3CA, PPP2R2A, RPS6KA3, RPS6, HIF1A, PRKAG1, EIF4EBP1, HMOX1, MTOR, AKT1, RHOD, PIK3C3, PIK3R2, RPS6KA2, VEGFB (includes EG:7423), PIK3C2A, EIF3H, EIF3F, EIF3J, PPP2R5A, PRKCI, EIF3I (includes EG:8668), PIK3CB, RPS6KA4, PPP2R5E, PPP2R1B, PRKCB |
| Docosahexaenoic Acid (DHA) Signaling | 0.0158 | RAC2, PIK3CA, AKT2, PIK3C2A, CASP3, BAD, PIK3R1, APAF1, RAC1, GSK3A, BAX, APP, RAC3, PIK3R3, BCL2L1, AKT1, PIK3C3, PIK3CB, PIK3CD, PIK3R2, GSK3B |
| HMGB1 Signaling | 0.0158 | MAP2K4, RAC2, RELA, PIK3CA, CDC42, PIK3R1, MAPK3, RAGE, HRAS, NFKB1, AKT1, HMGB1 (includes EG:3146), RHOB, CCL2, SP1, MAP2K2, RHOD, PIK3C3, PIK3R2, TNFRSF1B, IL8, MAP2K7, AKT2, MYST2, PIK3C2A, HAT1, RRAS, RAC1, IFNGR2, MAPK9, IL1R1, NFKB2, RAC3, PIK3R3, KAT2B, MAPK14, MYST3, RHOA, PIK3CB, MAP2K3, PIK3CD, ELK1, MAP2K5 |
| Glioma Signaling | 0.0174 | RAC2, PIK3CA, CAMK1D, PA2G4, MAPK3, PIK3R1, SUV39H1, SOS2, ABL1, CDKN2C, EGF, HRAS, RBL1, CCND1, SHC1, MTOR, AKT1, MAP2K2, PIK3C3, SOS1, E2F5, PIK3R2, E2F2, PDGFRB, EGFR, TP53, AKT2, RBL2, PRKCQ, CALM3, TFDP1, PIK3C2A, RRAS, RAC1, PLCG1, MDM2, RAC3, SIN3A, PIK3R3, PRKCI, E2F1, PIK3CB, PIK3CD, PRKCB |
| PDGF Signaling | 0.0209 | MAP2K4, PIK3CA, JAK1, PIK3R1, MAPK3, SOS2, SRF, ABL1, HRAS, INPPL1, SHC1, MAP2K2, PIK3C3, SOS1, CSNK2A1, PIK3R2, PDGFRB, SRC, PIK3C2A, RRAS, CRKL, MAP3K1, PLCG1, STAT3, INPP5D, PIK3R3, CSNK2A2, ABL2, PIK3CB, PIK3CD, EIF2AK2, ELK1, PRKCB |
| Neuregulin Signaling | 0.0224 | RAC2, BAD, MAPK3, PIK3R1, SOS2, EGF, HRAS, RPS6, SHC1, MTOR, AKT1, MAP2K2, HSP90AB1, SOS1, ERRFI1, PIK3R2, RNF41, EGFR, ITGB1, STAT5A, SRC, RPS6KB1, AKT2, PRKCQ, RRAS, CRKL, ITGA2, RAC1, PLCG1, HBEGF, ERBB3, ITGA3, RAC3, PIK3R3, ERBB2IP (includes EG:55914), PRKCI, PTPN11, TGFA, ELK1, PRKCB |
| JAK/Stat Signaling | 0.0234 | RAC2, PIK3CA, JAK1, PIAS2, PIK3R1, MAPK3, SOS2, HRAS, SHC1, MTOR, AKT1, MAP2K2, PIK3C3, SOS1, PIK3R2, STAT5A, PTPN6, AKT2, PIK3C2A, RRAS, RAC1, SOCS4, STAT3, RAC3, PIK3R3, PIAS4, PTPN11, PIK3CB, PIK3CD, SOCS5 |
| Melanoma Signaling | 0.0269 | TP53, RAC2, AKT2, PIK3CA, PIK3C2A, BAD, RRAS, PIK3R1, MAPK3, RAC1, HRAS, MDM2, RAC3, CCND1, PIK3R3, AKT1, MAP2K2, PIK3C3, E2F1, PIK3CB, PIK3CD, PIK3R2 |
| Endoplasmic Reticulum Stress Pathway | 0.0282 | TRAF2, CASP3, ERN1, XBP1, ATF4, MAP3K5 (includes EG:4217), HSPA5, EIF2AK3, CASP7, TAOK3, MBTPS2 |
| Estrogen Receptor Signaling | 0.0347 | TAF4B, TAF11, POLR2F, MAPK3, SOS2, POLR2J, HRAS, POLR2B, TAF13, MNAT1, GTF2A1, SMARCA4, EP300, SHC1, DDX5, GTF2B, POLR2C, NCOA2, PCK2, MAP2K2, MED15, SOS1, TAF3, TAF2, GTF2H3, PRKDC, HIST3H3 (includes EG:8290), SRC, MED1, RRAS, TAF15, TAF5L, TBP, RBM9, MED14, KAT2B, TAF6L, HDAC3, TAF1, H3F3A (includes EG:3020), TAF4, GTF2E1, ERCC3, NCOA1, GTF2H1, NRIP1, TRRAP, CARM1 |
| Ceramide Signaling | 0.0355 | MAP2K4, RAC2, RELA, PIK3CA, BAD, PPP2R2A, PIK3R1, MAPK3, PPP2R5B, HRAS, NFKB1, CTSD, AKT1, PIK3C3, PIK3R2, TNFRSF1B, SMPD3, NSMAF, AKT2, S1PR2, PIK3C2A, RRAS, MAP3K1, RAC1, CERK, NFKB2, RAC3, PPP2R5A, S1PR4, PIK3R3, PPP2R4, PIK3CB, PIK3CD, PPP2R5E, DIABLO, PPP2R1B |
| IL-4 Signaling | 0.0417 | RAC2, PIK3CA, JAK1, PIK3R1, SOS2, HLA-DRB1, HRAS, INPPL1, SHC1, MTOR, NFAT5, AKT1, PIK3C3, HLA-DRA, SOS1, PIK3R2, RPS6KB1, PTPN6, IL4R, AKT2, PIK3C2A, IL13RA1, RRAS, HMGA1, RAC1, RAC3, INPP5D, PIK3R3, PIK3CB, PIK3CD, HLA-DRB5 |
| CTLA4 Signaling in Cytotoxic T Lymphocytes | 0.0447 | RAC2, AP2A1, PIK3CA, PPP2R2A, PIK3R1, CD4, CLTB, PPP2R5B, LCK, AKT1, PIK3C3, AP1S3 (includes EG:130340), PIK3R2, HLA-DPB1, AP2B1, PTPN6, AKT2, PIK3C2A, CLTC, RAC1, PLCG1, RAC3, PPP2R5A, AP2S1, AP1S1, PIK3R3, PTPN11, PPP2R4, SYK, LAT, ZAP70, FCER1G, CD86, PIK3CB, PIK3CD, PPP2R5E, PPP2R1B, HLA-DRB5, AP1G1 |
| LPS-stimulated MAPK Signaling | 0.0457 | MAP2K4, RELA, PIK3CA, CDC42, PIK3R1, MAPK3, SRF, HRAS, MAP3K5 (includes EG:4217), NFKB1, PAK1, MAP2K2, MAP3K7, PIK3C3, PIK3R2, CHUK, PRKCQ, PIK3C2A, ATF1, RRAS, RAC1, MAPK9, NFKB2, ATF2, PIK3R3, MAPK14, PRKCI, MAP2K3, PIK3CB, PIK3CD, ELK1, PRKCB |
| CD28 Signaling in T Helper Cells | 0.0468 | MAP2K4, RAC2, RELA, PIK3CA, CDC42, ARPC1B, CD4, PIK3R1, NFKB1, PTPRC, LCK, PAK1, NFAT5, ACTR3, AKT1, PPP3CB, MAP2K2, PPP3R1, PIK3C3, ARPC3, CHUK, PIK3R2, HLA-DPB1, PPP3CA, AKT2, PTPN6, PRKCQ, CALM3, PIK3C2A, MAP3K1, RAC1, MAPK9, PLCG1, PPP3CC, NFKB2, RAC3, PIK3R3, PTPN11, SYK, ARPC2, LAT, ZAP70, FCER1G, CD86, VAV1, PIK3CB, PIK3CD, HLA-DRB5 |

**Supplementary Table S6: Base pairs determined to be in different copy number states in the initial tumor analysis and in the drug resistant tumor recurrence.**

|  | Initial Tumor | Tumor Recurrence |
| --- | --- | --- |
| copy number loss | 745439900 | 864014009 |
| copy number neutral | 1428974388 | 1560292154 |
| copy number slight gain | 661593114 | 509495541 |
| copy number medium gain | 104480525 | 5107451 |
| copy number highest gain | 1688361 | 4068399 |

**Supplementary Table S7**

Regions where the copy number status changed from a loss to a gain between the initial tumor and the recurrence:

| 1 | 36563165 | 39058489 |
| --- | --- | --- |
| 1 | 121038131 | 142536046 |
| 3 | 116463833 | 120872385 |
| 6 | 58837268 | 62025536 |
| 14 | 102255919 | 106348275 |
| 18 | 16943155 | 18156372 |
| 18 | 19144435 | 21940312 |
| 18 | 22471758 | 76099875 |

Regions where the copy number changed from gain to loss:

| 1 | 58359310 | 58927463 |
| --- | --- | --- |
| 1 | 60485476 | 62086159 |
| 1 | 142536047 | 150238320 |
| 3 | 197503755 | 199353836 |
| 5 | 127127929 | 131227703 |
| 7 | 51328670 | 57948664 |
| 8 | 74039520 | 96238672 |
| 10 | 42137927 | 43519607 |
| 15 | 18822271 | 19219116 |
| 16 | 5909275 | 32354214 |
| 18 | 16774297 | 16943154 |
| X | 76669675 | 99338464 |

The observed increase of copy number loss regions was accompanied with a shift in the Loss of Heterogeneity (LOH) results.   Many of the regions that appeared incompletely LOH in the initial tumor had progressed to showing complete LOH.   The initial tumor showed 18.8% of the genome (564064734 bases) having the incomplete LOH signature.   This number dropped to 15.3% of the genome (459899307 bases) in the tumor recurrence,  contributing in part to the increase in the amount of the genome that showed complete LOH at 22.2% (667322815 bases), up from 5.1% (153585645 bases) in the original tumor.

|  | Tumor 1 | Tumor 2 |
| --- | --- | --- |
| Heterzygous | 2218079613 | 1763094467 |
| Incomplete LOH | 564064734 | 459899307 |
| Complete LOH | 153585645 | 667322815 |
